# Altered Blood and Brain Expression of Inflammation and Redox Genes in Alzheimer’s Disease, Common to APP^V717I^ × TAU^P301L^ Mice and Patients

**DOI:** 10.3390/ijms23105799

**Published:** 2022-05-21

**Authors:** Catalina Anca Cucos, Elena Milanesi, Maria Dobre, Ioana Andreea Musat, Gina Manda, Antonio Cuadrado

**Affiliations:** 1 Victor Babes National Institute of Pathology, 050096 Bucharest, Romania; anca.cucos@ivb.ro (C.A.C.); elena.milanesi@ivb.ro (E.M.); maria.dobre@ivb.ro (M.D.); 2 Faculty of Medicine, Carol Davila University of Medicine and Pharmacy, 050474 Bucharest, Romania; ioana.a.musat@stud.umfcd.ro; 3 Department of Biochemistry, Medical College, Autonomous University of Madrid (UAM), 28049 Madrid, Spain; 4 Instituto de Investigaciones Biomédicas “Alberto Sols” (CSIC-UAM), 28029 Madrid, Spain; 5 Instituto de Investigación Sanitaria La Paz (IdiPaz), 28046 Madrid, Spain; 6 Centro de Investigación Biomédica en Red de Enfermedades Neurodegenerativas (CIBERNED), 28029 Madrid, Spain

**Keywords:** Alzheimer’s disease, gene expression, inflammation, redox alterations, hippocampus, blood

## Abstract

Despite intensive research, the pathophysiology of Alzheimer’s disease (AD) is still not fully understood, and currently there are no effective treatments. Therefore, there is an unmet need for reliable biomarkers and animal models of AD to develop innovative therapeutic strategies addressing early pathologic events such as neuroinflammation and redox disturbances. The study aims to identify inflammatory and redox dysregulations in the context of AD-specific neuronal cell death and DNA damage, using the APP^V717I^× TAU^P301L^ (AT) mouse model of AD. The expression of 84 inflammatory and 84 redox genes in the hippocampus and peripheral blood of double transgenic AT mice was evaluated against age-matched controls. A distinctive gene expression profile in the hippocampus and the blood of AT mice was identified, addressing DNA damage, apoptosis and thrombosis, complemented by inflammatory factors and receptors, along with ROS producers and antioxidants. Gene expression dysregulations that are common to AT mice and AD patients guided the final selection of candidate biomarkers. The identified inflammation and redox genes, common to AD patients and AT mice, might be valuable candidate biomarkers for preclinical drug development that could be readily translated to clinical trials.

## 1. Introduction

Alzheimer’s disease (AD) is a chronic and progressive neurodegenerative disorder, characterized by broad neurologic, cognitive, functional, behavioral and psychological impairments [1,2], that is considered the greatest challenge for health and social care in the 21st century, having a global impact on patients and their families, communities and society [3].

Genetic factors underlie AD pathophysiology, with gene variants of *APP*, *PSEN1* and *PSEN2* leading to early AD onset (EOAD) in familial forms of the disease, and the APOE ε4 allele being associated with increased disease severity in EOAD as well as in late-onset forms (LOAD) of familial and sporadic AD [4,5]. While genetics provides a solid background for disease onset and evolution, the dysregulation of gene circuits and functional impairment of critical proteins are intensively investigated as well to obtain new insights into AD’s pathophysiology [6,7].

Nevertheless, despite decades of intensive research in the field, AD’s pathophysiology is still not fully understood, and currently there are no effective treatments. For instance, clinical trials on drugs addressing amyloid plaques and TAU tangles, the typical pathologic features of AD, have failed to slowdown cognitive decline in patients with mild cognitive impairment or AD dementia [8,9,10]. Therefore, AD research has currently shifted to alternative pathological processes that may initiate and further sustain neuronal cell death and the accumulation of DNA damage in the hippocampus [11,12,13]. Evidence is continuously being produced that systemic and local inflammation and redox alterations precede overt AD symptoms by years, and may be considered at least risk factors, if not straightforward mechanisms in AD pathogenesis [14]. Thus, neuroinflammation mediated by resident cells (microglia and astrocytes) as well as by capillary endothelial cells and brain-infiltrating leukocytes, in conjunction with chronic alterations of redox-sensitive signaling pathways [15,16], were shown to greatly contribute to synaptic dysfunction, neuronal death and the inhibition of neurogenesis [17,18]. Upon DNA damage, microglia and astrocytes accumulate DNA fragments “leaking” to the cytoplasm where they trigger STING-dependent sterile inflammatory processes and neurotoxicity [19]. Moreover, oxidative DNA damage [20,21] in the AD brain, tightly connected with Aβ and TAU pathologies [12], seem to occur prior to disease onset [22,23].

A case–control clinical study recently published by us [24] evidenced in whole blood nine inflammation and seven redox genes that discriminate very well between mild AD patients and controls, resulting from the increased activity of NFκB, NRF2 and several zinc finger and helix-loop-helix transcription factors. Altogether, the results emphasize systemic dysfunctions that might be an echo of the pathological events occurring in the AD brain.

In addition to new inflammation and redox biomarkers, appropriate animal models of AD that recapitulate the combined human amyloid and TAU pathology and express inflammation- and redox-related biomarkers, are highly needed for the preclinical development of anti-inflammatory and antioxidant therapeutic strategies in AD [25].

In this context, we carried out a case–control study investigating through qRT-PCR the expression levels of 168 inflammation and redox genes in the hippocampus and peripheral whole blood of double transgenic (hAPP^V717I^ and hTAU^P301L^) mice against age-matched controls. These mice, which express APP and TAU variants of human genes in the brain [26], have been shown to recapitulate with aging the combined amyloid and TAU pathology of the human disease [26,27]. The study highlighted inflammation and redox disturbances in the context of neuronal cell death and DNA damage, and allowed the selection of particular genes that are commonly dysregulated in AD patients and the investigated mouse model. This gene panel may be further used for the preclinical development of innovative anti-inflammatory and antioxidant therapies in AD.

## 2. Results

The expression levels of 168 inflammation and redox genes were analyzed by qRT-PCR using pathway-focused arrays in the hippocampus and whole blood from AT mice developing amyloidopathy (APP^V717I^) and tauopathy (TAU^P301L^), in comparison with age-matched WT controls. Gene expression levels were calculated as 2^−∆CT^ values that were further processed as fold change (FC) values. Results were presented as fold regulation (FR) values, which are equal to the FC value if the FC is higher than 1 (resulting in positive superunit values for over-expressed genes), and is calculated as the negative inverse of the FC when the FC is below 1 (resulting negative superunit values for under-expressed genes).

### 2.1. Gene Expression Changes in the Hippocampus of AT Mice

**Figure 1:**
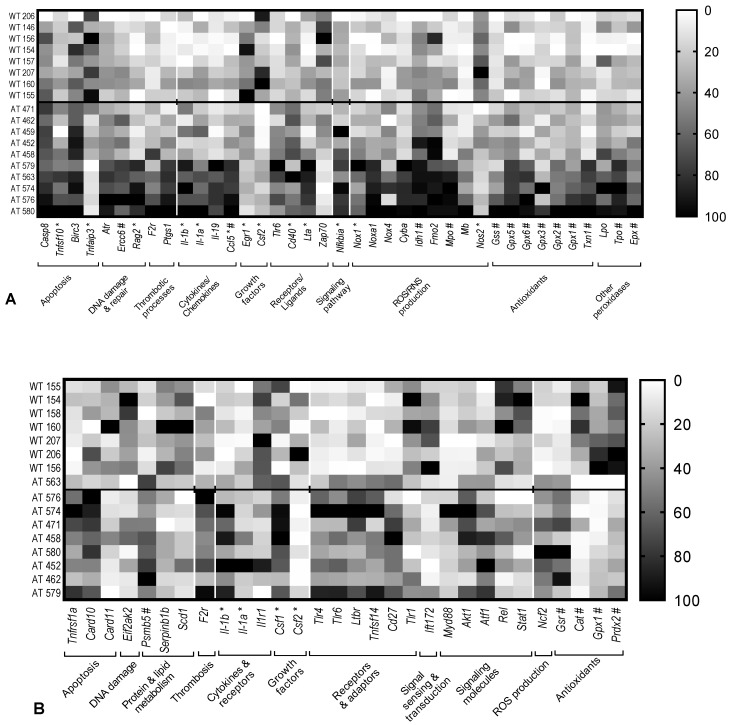
Heat maps of individual gene expression data in the hippocampus of 10 transgenic AT mice and 8 WT controls (**A**) and in the whole blood of 9 AT mice and 7 WT controls (**B**). Genes with |FR| > 1.5 and *p* < 0.05 in AT mice vs. WT controls are shown. Data are presented as 2^−ΔCT^ values and are scaled considering the highest value as 100%. Genes that are over-expressed in AT mice appear in dark tones in this group and in lighter tones in the control group. Genes that are under-expressed in AT mice appear in dark tones in the control group and lighter tones in diseased mice. NFkB target genes are marked with *, while redox-responsive genes are marked with #.

We analyzed first those genes that were differentially expressed with the FR threshold set at 1.5 (*p* < 0.05). Thirteen inflammatory genes were over-expressed in the hippocampus of AT mice as compared with WT controls, while four genes were down-regulated (*Tnfaip3*, *Erg1*, *Csf2* and *Zap70*), as presented in Figure 1A. Some of these genes underlie basic pathological processes in the AD brain, such as apoptosis (*Casp8*, *Tnfsf10*, *Birc3* and *Tnfaip3*) and DNA damage responses (*Atr*, *Ercc6* and *Rag2*). Thrombotic processes were also emphasized at gene expression level (*F2r* and *Ptgs1*) in the hippocampus of AT mice. Other genes identified in this study address the immune response by encoding cytokines (*Il1b*, *Il1a, Il19* and *Lta*), chemokines (*Ccl5*) and growth factors (*Erg1* and *Csf2*), as well as inflammation-triggering receptors (*Tlr6* and *Cd40*) or signaling adaptors (*Zap70*). Most of these genes are involved in NFkB signaling or are NFkB target genes (marked with * in Figure 1A), demonstrating that NFkB-mediated inflammation has a broad fingerprint in the hippocampus of AT mice.

Twenty-one transcripts of redox genes were found to be up-regulated in the hippocampus of AT mice compared to WT controls (Figure 1A), and only one gene showed decreased expression (*Nos2*). Some of the identified genes participate in the production of reactive oxygen species (*Nox1*, *Noxa1*, *Nox4*, *Cyba*, *Idh1*, *Fmo2*, *Mpo* and *Mb*) or reactive nitrogen species (*Nos2*). In turn, other genes identified in this study encode antioxidants that are involved in glutathione biosynthesis (*Gss*) and metabolism (*Gpx1*, *Gpx2, Gpx3*, *Gpx5* and *Gpx6*), or in the thioredoxin system (*Txn1*). Several peroxidases (*Mpo, Lpo*, *Tpo* and *Epx*) were found to be up-regulated as well. Part of the identified genes are redox-responsive genes (marked with # in Figure 1A), with their increased transcription consequently suggesting an enhanced oxidative activity in the hippocampus of AT mice.

### 2.2. Gene Expression Changes in the Blood of AT Mice

A relatively different pattern of gene expression changes was registered in the blood of AT mice as compared to WT controls (Figure 1B). We identified fifteen up-regulated and ten down-regulated genes related to the inflammatory NFkB pathway. These genes are involved in apoptosis (up-regulated *Tnfrsf1a* and *Card10*, and down-regulated *Card11*), DNA damage (down-regulated *Eif2ak2*) and thrombosis (up-regulated *F2r*), along with alterations in the metabolism of proteins (up-regulated *Psmb5)* and lipids (down-regulated *Serpinb1b* and *Scd1*). As in the hippocampus, we identified in blood the dysregulation of several genes involved in immune responses. These genes encode cytokines and their receptors (up-regulated *Il1a* and *Il1b*, and down-regulated *Il1r1*), and growth factors (up-regulated *Csf1* and down-regulated *Csf2*). Moreover, we identified the increased expression of genes related to NFkB or to interfering signaling pathways, encoding receptors and ligands (up-regulated *Tlr4*, *Tlr6*, *Ltbr* and *Cd27*, and down-regulated *Tlr1*), ligands (up-regulated *Tnfsf14*)*,* down-stream adaptor molecules(up-regulated *Myd88* and down-regulated *Ift172*) and kinases (up-regulated *Akt1*), as well as transcription factors (up-regulated *Rel*, *Atf1* and *Stat1*). The activation of the pro-inflammatory NFkB pathway is further sustained by the over-expression of NFkB target genes in the blood of AT mice (marked with * in Figure 1B), namely *Il1b*, *Il1a*, *Csf1* and *Csf2*.

Five redox genes were found to be dysregulated in the blood of AT mice compared to WT controls (Figure 1B). They are involved in superoxide production (up-regulated *Ncf2*) and antioxidant mechanisms (up-regulation of *Gsr*, accompanied by down-regulation of *Gpx1*, *Cat* and *Prdx2*), indicating redox disturbances in the blood of AT mice.

### 2.3. Analysis of Inflammation and Redox Gene Changes with Higher FR Values

**Figure 2:**
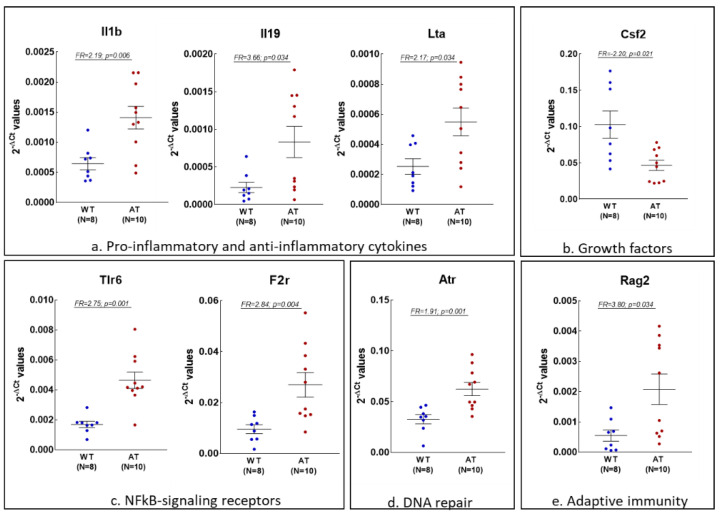

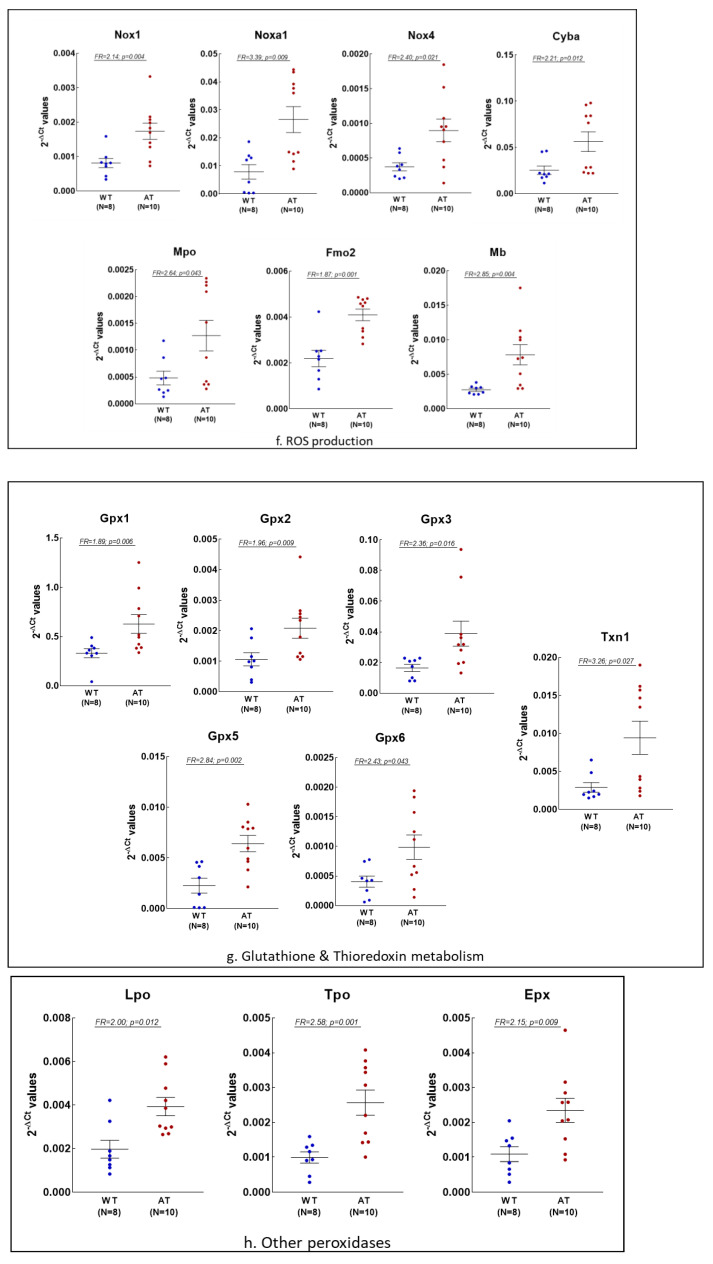
Inflammation and redox-related genes that are differentially expressed in the hippocampus of AT mice (N = 10) vs. WT mice (N = 8), exhibiting |FR| values > 1.8. Data are presented as 2^−∆CT^ values, and lines represent the expression average ± standard error of the mean (SEM). Comparisons between mice groups were made using the Mann–Whitney U-test, and differences were considered significant for *p* < 0.05.

**Figure 3:**
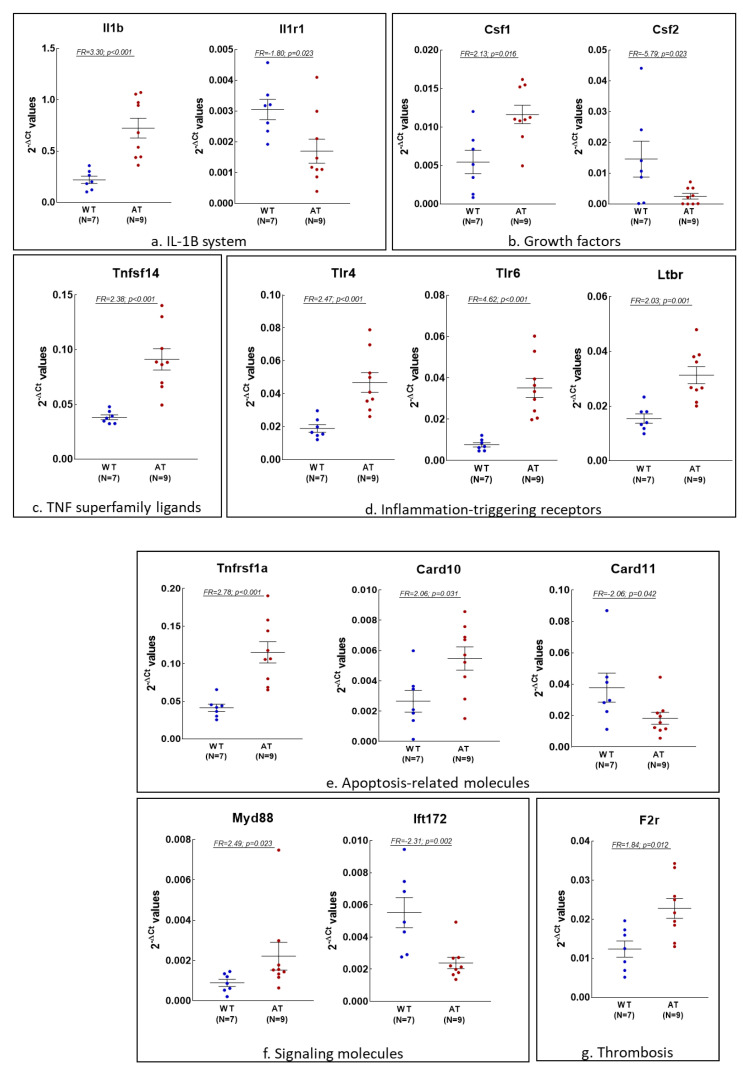

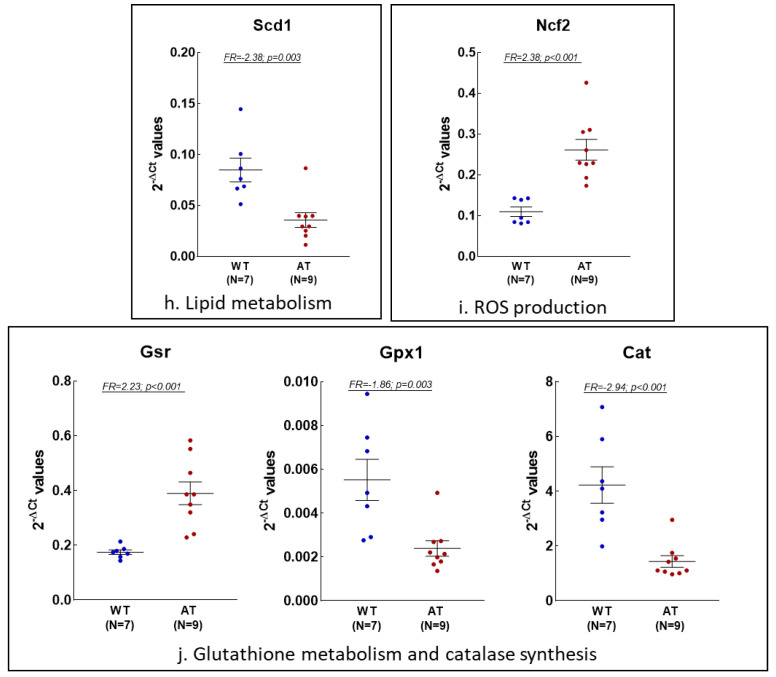
Inflammation and redox-related genes differentially expressed in the whole blood of AT mice (N = 9) vs. WT mice (N = 7), exhibiting |FR| values > 1.8. Data are presented as 2^−∆CT^ values, and lines represent the expression average ± standard error of the mean (SEM). Comparisons between mice groups were made using the Mann–Whitney U-test, and differences were considered significant for *p* < 0.05.

Considering that genes with higher FR values are supposed to capture biologically meaningful information, we further selected those inflammation and redox genes exhibiting |FR| values > 1.8 and (*p* < 0.05) in the hippocampus and blood of AT mice as compared to WT controls (Figure 2 and Figure 3, respectively).

In the hippocampus of AT mice, we identified seven up-regulated genes (Figure 2a,c–e) that encode cytokines (Figure 2a: the pro-inflammatory *Il1b* and *Lta*, along with the anti-inflammatory *Il19*) or growth factors (Figure 2b: *Csf2*), NFkB signaling receptors (Figure 2c: *Tlr6* and *F2r*), molecules involved in DNA repair (Figure 2d: *Atr*) or gene rearrangement in antigen receptors specifically expressed by B or T lymphocytes (Figure 2e: *Rag2*). In the array of inflammation genes, only *Csf2*, encoding the granulocyte-monocyte colony-stimulating factor (GM-CSF) was under-expressed (Figure 2b). We also found 16 up-regulated redox genes fulfilling the same criteria in the hippocampus of AT mice (Figure 2f–h). Parts of these genes encode molecules involved in ROS production (Figure 2f), such as NADPH-oxidase components (*Nox1*, *Noxa1*, *Nox4* and *Cyba*) and myeloperoxidase (*Mpo*), along with the NADPH-dependent dimethylaniline monooxygenase (*Fmo2*) and the myoglobin oxygen transporter (*Mb*). The transcript levels of some antioxidant genes were also found to be elevated (Figure 2g), comprising genes that encode glutathione peroxidases (*Gpx1, 2, 3, 5* and *6*) or thioredoxin (*Txn1*). In addition, three genes encoding peroxidases other than myeloperoxidase and glutathione peroxidases were found to be up-regulated as well (Figure 2h: *Lpo*, *Tpo* and *Epx*).

In the blood of AT mice, we identified 15 inflammation genes with |FR| values > 1.8 (*p* < 0.05), related to the NFkB signaling pathway (Figure 3a–h), that were all up-regulated, excepting *Csf2*and *Scd1*. These genes encode pro-inflammatory interleukins and their receptors (Figure 3a: *Il1b* and *Il1r1*, respectively), growth factors (Figure 3b: *Csf1* and *Csf2*) or ligands belonging to the TNF superfamily (Figure 3c: *Tnfsf14*), along with inflammation-triggering receptors (Figure 3d: *Tlr4*, *Tlr6* and *Ltbr*), apoptosis-related factors (Figure 3e: *Tnfrsf1a*, *Card10* and *Card11*) and signaling molecules (Figure 3f: *Myd88* and *Ift172*). In addition, we found the up-regulated *F2r* gene is involved in thrombosis (Figure 3g), and the down-regulated *Scd1* gene is related to monounsaturated fatty acid biosynthesis (Figure 3h). As well as inflammatory genes, we found four up-regulated redox genes in the blood of AT mice that are involved in superoxide production (Figure 3i: *Ncf2*) or in antioxidant responses that address glutathione metabolism (*Gsr* and *Gpx1*) and catalase synthesis (*Cat*) (Figure 3j), with *Gpx1* and *Cat* being involved in hydrogen peroxide detoxification.

### 2.4. Hippocampus–Blood Comparison

**Figure 4:**
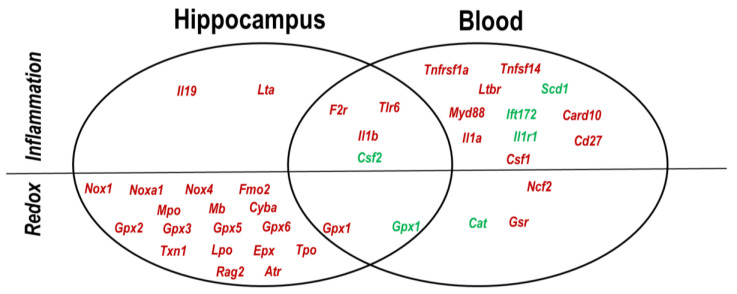
Inflammation and redox-related genes differentially and commonly expressed in the hippocampus and in the whole blood of AT mice vs. WT controls. Only genes with |FR| > 1.8 and *p* < 0.05 are reported. Red font indicates up-regulated and green font down-regulated genes.

In the pool of the dysregulated genes described in Figure 2 and Figure 3, exhibiting |FR| values > 1.8 (*p* < 0.05), five genes had expression changes both in the hippocampus and whole blood of AT mice as compared to WT controls (Figure 4). Thus, the pro-inflammatory genes *F2r*, *Tlr6* and *Il1b* were found to be up-regulated, while *Csf2* was down-regulated. Accordingly, some inflammatory changes detected in the brain of AT mice seem to have an echo in peripheral blood. Meanwhile, the antioxidant *Gpx1* gene presented opposite trends, being up-regulated in the hippocampus and down-regulated in blood, possibly due to oxidative status differences in the brain and blood of AT mice. Indeed, a distinctive and broad inflammatory NFkB fingerprint was evidenced in blood, whereas wide-ranging redox changes occurred specifically in the hippocampus of AT mice (Figure 4), suggesting that distinctive pathological processes take place in each of these compartments.

### 2.5. Correlation of Gene Expression Levels with Age

**Figure 5:**
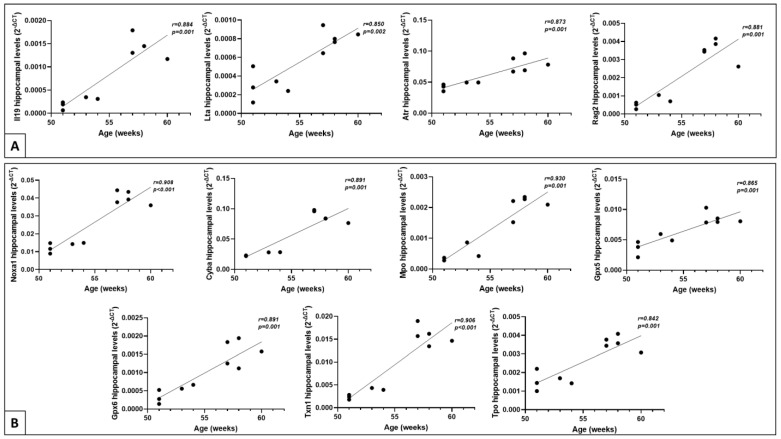
Correlations between the mRNA hippocampal levels of (**A**) inflammation and (**B**) redox genes with the age (in weeks) of AT mice (N = 10). Genes with |FR| > 1.8 and *p* < 0.05 were analyzed. The gene expression levels are presented as 2^−∆CT^ values.

The analysis of inflammatory and redox gene dysregulation as a result of aging was analyzed in AT mice. A correlation study between age and the significantly dysregulated genes detected in the hippocampus (see Figure 2) and in the blood (see Figure 3) of AT mice and WT controls was performed. While no statistically relevant correlations were detected in the blood of AT mice (data not shown), eleven genes were significantly correlated with age (r > 0.800, *p*< 0.05) exclusively in the hippocampus of AT mice (Figure 5). Four inflammation genes correlated with age in the AT group (Figure 5A), namely the anti-inflammatory *Il19* gene and the pro-inflammatory *Lta* gene, both encoding cytokines, along with the *Atr* gene involved in DNA repair and the *Rag2* gene implicated in V(D)J gene recombination during B and T cell development. More correlations with age were registered in the case of hippocampal redox genes. These genes (Figure 5B) address ROS production, either superoxide (*Noxa1* and *Cyba)* or hypochlorous acid (*Mpo*) generation, along with antioxidants such as glutathione peroxidases (*Gpx5* and *Gpx6*), thioredoxin (*Txn1*) and thyroid peroxidase (*Tpo*). The lack of correlation in the control WT group in fact suggests that the associations observed in the hippocampus of AT mice reflected disease progression with age rather than biological aging. Thus, increased expression of the mentioned genes is expected to appear in more advanced forms of AD, as detected by us in mice older than 55 weeks.

### 2.6. Comparison of Dysregulated Inflammation and Redox Genes in AT Mice vs. AD Patients

We further compared the expression of genes identified in the hippocampus of AT mice (Figure 2), with |FR| > 2 and *p* < 0.05, with available microarray datasets of post-mortem brain samples from AD patients (Supplementary Table S4). As shown in Table 1, several dysregulated genes in AD patients were also altered in the AT mice model, including *Il19, F2r, Tlr6, Il1b, Lta, Csf2, Gpx3 and Nox1*. This panel of genes might be meaningful for preclinical investigations, using AT mice as a reliable model to monitor inflammatory and redox changes that could have a rapid translation into clinical studies.

Genes with modified expression in the blood of AT mice (Figure 3), with FR > 1.8 (*p* < 0.05), were compared with previous data obtained by us in the blood of mild AD patients [24]. Four inflammation genes and one redox gene were found to be over-expressed in the blood of both AT mice and mild AD patients (Table 2). The gene panel comprises inflammation genes related to the NFkB signaling pathway, encoding receptors (*Ltbr* and *F2r*), cytokines (*I11b*) and growth factors (*Csf1*), along with the antioxidant *Gsr* gene.

The transcript levels of *Gsr*, identified using PCR arrays in the blood of 9 AT mice vs. 7 WT controls, with a mean age of 54.6 ± 3.5 weeks (Figure 3), as well as in the blood of mild AD patients [24], were validated in independent mice groups of 21 AT vs. 12 WT controls, with a mean age of 48.5 ± 4.9 weeks. Moreover, younger animals (7 AT vs. 8 WT controls, with a mean age of 37.1 ± 0.8 weeks), supposed to have a milder form of disease [27], were also investigated. In addition to the antioxidant *Gsr* gene, we analyzed in parallel the *Osgin1* gene as a marker of enhanced oxidative activity, considering that only a relatively limited fingerprint of redox alterations was detected in the blood of AT mice (Figure 4).

The antioxidant *Gsr* gene and the redox-sensitive *Osgin1* gene were both up-regulated in the group of 49-week-old mice and in the group of 37-week-old mice (Table 3). Results indicate an enhanced oxidative activity in the blood of AT mice that persisted during disease evolution and induced enhanced *Gsr* expression for antioxidation. Considering that *Gsr* is a target of the cytoprotective NRF2 transcription factor [28], it appears that NRF2 might become activated in response to an enhanced oxidative activity in the blood leukocytes of AT mice and mild AD patients. Altogether, the comparison of gene expression in the blood of AT mice and mild AD patients emphasized glutathione reductase as an early blood redox biomarker that can be evidenced even in milder forms of the disease, both in patients and AT mice.

## 3. Discussion

Clinical interventions in AD would greatly benefit from the detection of genetic blood biomarkers that would help in disease prognosis, monitoring and drug response. Accordingly, this study was aimed at identifying comparatively relevant biomarkers related to altered gene expression in the hippocampus and blood of the double transgenic AT mouse model. These mice express the mutated *APP* and *TAU* human genes in the brain, and closely recapitulate the human amyloid and TAU pathology [26], including cognitive deficits and the alteration in exploratory and anxiety-like behavior, fear learning and inflexibility in hippocampus-dependent learning [1,29].

The molecular fingerprint of inflammation and redox disturbances was characterized using pathway-focused PCR arrays in the context of disease-specific neuronal cell death and DNA damage. A broad molecular signature of NFkB-mediated inflammation and redox alterations was highlighted in the hippocampus and blood of AT mice, probably accounting for the pathologic features of AD in the investigated mouse model. As will be discussed below, several of the gene expression changes evidenced in AT mice were also detected in datasets on post-mortem brain samples from AD patients, further supporting their relevance in translational medicine.

In the hippocampus of AT mice, modified expression of genes involved in basic pathologic features of AD was registered, addressing DNA damage [30,31,32], TRAIL/caspase 8-mediated apoptosis [33] and increased risk of thrombosis [34]. Rescue mechanisms against apoptosis and DNA damage were highlighted by the over-expression of dedicated repair genes, representing an indirect proof of ongoing deleterious processes. Nevertheless, these mechanisms do not seem to be efficient enough for repairing cellular damage, as neurodegeneration was shown to evolve with age in AT mice [27].

Neuroinflammation is a central mechanism in AD, which exacerbates the amyloid and TAU pathology [14]. The transcription factor NFkB is considered a primary regulator of inflammatory responses in the AD brain, its activation being observed in microglia and astroglia surrounding Aβ plaques [35]. In this context, our results emphasized the up-regulation of the *Tlr6*and *Lta* genes, known to elicit NFkB-driven inflammation. In AD patients (Table 1), *TLR6* and *LTA* were up-regulated in different brain regions, including the frontal cortex (*TLR6* and *LTA*), gray matter and neocortex (*LTA*). NFkB activation and the enhanced production of pro-inflammatory factors were additionally demonstrated in the present study by the over-expression of various NFkB target genes. For instance, we found over-expression of genes encoding the pro-inflammatory cytokines IL-1α and IL-1β in the hippocampus of AT mice. IL-1β, a key regulator of neuroinflammation, has been reported to surround amyloid plaques in AD patients, and to be involved in excessive production and processing of the amyloid protein precursor [36,37]. IL-1β levels were found to be elevated in the brains of AD patients along with a six-fold increase in IL-1β immunoreactive microglia in the cerebral cortex [38]. However, the results obtained in various studies are contrasting [39,40,41]. For instance, the analysis of the GSE28146 dataset (Table 1) showed that *IL1B* mRNA levels were down-regulated in the grey matter of seven AD patients with a severe form of disease. Along with the pro-inflammatory *Il1b* gene, the up-regulation of the anti-inflammatory *Il19* gene [42] was detected in the hippocampus of AT mice, as well as in the frontal cortex of AD patients (Table 1). The observation that hippocampal *Il19* gradually increased during AD progression in APP/PS1 Tg transgenic mice [43] sustains our findings.

The only markedly down-regulated inflammatory gene in the hippocampus of AT mice was *Csf2* that encodes GM-CSF, an important neurotrophic factor of the central nervous system [44]. A recent study performed on mild to moderate AD patients showed that treatment with GM-CSF provided memory-enhancing benefits [45], and a clinical trial (NCT04902703) on AD patients for evaluating the safety and efficacy of GM-CSF (Sargramostim) is ongoing. In addition, a marked reduction in the GM-CSF receptor was detected in the hippocampus, suggesting a broader contribution of GM-CSF signaling to AD pathology [44].

As well as the dysregulation of innate immune responses, the impairment of adaptive immunity was attested in the hippocampus of AT mice, pointing towards an anergic state of T lymphocytes [46,47]. This could be a compensatory mechanism for controlling neuroinflammatory responses to Aβ aggregates and other toxic molecules in AD [48]. In turn, the increased levels of the RANTES chemokine reported in this study indicate the enhanced recruitment of T lymphocytes in the AD brain, which may sustain perivascular inflammation [49] and provide protection against thrombin toxicity [50].

A distinctive redox status was evidenced at the transcriptional level in the hippocampus of AT mice, characterized by the over-expression of genes involved in ROS production, mainly addressing superoxide generation by NOX1 [51]. *NOX1* transcript levels were found to be up-regulated in the hippocampus of AD patients (Table 1), as well as in the frontal lobe of AD patients in the early stages of disease [52]. Moreover, a post-mortem study on the frontal and temporal cortex from mild cognitive impairment and AD patients in different stages showed elevated levels of various NOX components [53]. In addition to NOX enzymes, increased transcript levels of *MPO* were found in human AD brains, suggesting a potential contribution of hypochlorous acid to oxidative damage in AD [54].

The only redox gene found to be down-regulated in the hippocampus of AT mice was *Nos2* which is involved in nitric oxide (NO) production [55]. Due to the duality of NO, data on its role in AD are contradictory [56]. It has been shown that genetic removal of *Nos2* can promote TAU pathology [55], hence sustaining other studies that emphasize the protective role of NO in AD [55,57]. In turn, other studies suggest that NO is involved in nitrosative damage in AD [58,59] via the generation of the highly toxic peroxynitrite in the presence of superoxide [59].

The over-expression of several genes involved in ROS production was accompanied by elevated transcript levels of particular antioxidant genes involved in glutathione biosynthesis and metabolism or in the thioredoxin system, indicating that protective antioxidant mechanisms might be elicited in the AD brain in response to an enhanced oxidative activity. In line with our data, increased levels of the *GPX3* transcript were found in the entorhinal cortex from 36 AD patients compared to 16 controls (Table 1). Moreover, thioredoxin up-regulation was shown to play a neuroprotective role in AD [60]. Nevertheless, the antioxidant defense appears to be inefficient as long as disease is persisting and progressing with age in AT mice. It has been suggested that the dysregulation of glutathione homeostasis may contribute to AD pathogenesis [61], and that glutathione levels assessed by proton magnetic resonance spectroscopy in specific brain regions could be clinically relevant in AD [62].

Some of the genes found to be dysregulated in the hippocampus of AT mice were found to be dependent on disease progression with age, therefore being suitable for evaluating the impact of experimental therapies on the disease course.

Blood biomarkers would be a valuable tool for early diagnosis and disease monitoring using minimally invasive methods, if AD has an echo in the blood [63]. We highlighted a common gene transcription pattern in the hippocampus and whole blood of AT mice, suggesting that some transcriptional changes in the brain are mirrored in peripheral blood where they can be dynamically monitored. Such a gene is *Il1b* whose product was also significantly elevated in the blood of AD patients [64]. In addition, our results indicate that there are important differences in the ongoing pathologic and repair processes occurring in the hippocampus and in the blood of AT mice, with redox disturbances being dominant in the hippocampus, while the inflammation fingerprint is dominant in the blood of AT mice.

By comparing the expression pattern of inflammation and redox genes in AT mice and AD patients, we selected a panel of common genes, specific either for brain or blood.

The hippocampus panel comprises the inflammatory genes *F2r*, *Tlr6* and *Lta,* participating in the NFkB signaling pathway, along with the anti-inflammatory *Il19* gene, complemented by the redox genes *Nox1* and *Gpx3* that are involved in superoxide production and hydrogen peroxide detoxification, respectively. Nonetheless, other genes found by us to have a significant expression change in AT mice might also be useful for preclinical investigations, considering that their products were shown in various human and mice studies to have a role in AD pathogenesis.

In blood, we identified a distinctive panel of inflammatory genes that encode receptors (*Ltbr* and *F2r*), cytokines (*Il1b*) and growth factors (*Csf1*), along with the *Gsr* redox gene. *Gsr* appears to be an early blood biomarker that can be evidenced even in milder forms of the disease, both in patients and AT mice. Of note is that *F2r* over-expression is common to the hippocampus and blood, indicating endothelial dysfunction and an increased risk of thrombosis in these compartments, both in mice and AD patients. Moreover, non-canonical NFkB activation through the lymphotoxin B receptor encoded by *Ltbr* was also demonstrated both in the hippocampus and blood of AT mice.

Altogether, the obtained results bring into focus panels of genes with a common trend of expression changes in AT mice and AD patients that may overcome the generally low animal-to-human translational success [65]. Accordingly, the double transgenic AT model, relevant for mild to moderate AD, might be useful for drug development by allowing a rapid and reliable transfer of preclinical results into clinical studies. Moreover, those common genes with similar expression changes in the blood and hippocampus of AT mice would be useful for dynamic therapy monitoring in blood samples in which the echo of the disease was evidenced at the level of particular inflammation and redox genes.

## 4. Materials and Methods

### 4.1. Animal Model

As animal model of AD, we used a double transgenic mouse model with neuronal expression of human hAPP^V717I^ and hTAU^P301L^proteins (hereinafter referred to as AT) generated in C57/BL6j background. These mice express both APP and TAU human genes, under the control of the mouse *Thy1* gene promoter. The AT mice were obtained by crossing heterozygous APP^V717I^mice with homozygous TAU^P301L^ mice, for more than eight generations, as described previously [26]. The characteristics of the APP^V717I^ and TAU^P301L^ transgenic mice have been previously described [27,29],recapitulating with aging a combined amyloid and TAU pathology. In this model, the amyloid pathology sets in at 10 to 12 months, and tauopathy is notable at approximately 13 months [27].

Hippocampus and whole blood from 10 transgenic AT mice and 8 age-matched wild type mice (WT), with a mean age of 54.4 ± 3.4 weeks were investigated using pathway-focused PCR arrays. Additionally, the *Gsr* and *Osgin1* genes were analyzed by qPCR single gene assay in two independent groups, the first one comprising 21 AT vs. 12 WT (mean age 48.5 ± 4.9 weeks) and the second one with 7 AT vs. 8 WT mice (mean age 37.1± 0.8 weeks).

Mice were group-housed in simple cages under standard conditions (normal 12 h light/dark cycle, constant temperature and humidity), with ad libitum access to food and water. All animal experiments were approved by the Ethics Committee of “Victor Babes” National Institute of Pathology, Bucharest, Romania, authorization no. 39/11.04.2017 and no. 91/30.07.2021, and by the Romanian National Authority for Veterinary Research, authorization no. 385/9.02.2018 and no. 648/10.09.2021. Experiments were carried out according to the European Directive 2010/63/EU.

### 4.2. Blood and Hippocampi Collection

Blood was collected by retro-orbital puncture in PAXgene RNA stabilizer solution (Qiagen, Hilden, Germany). Prior to brain collection, mice were anesthetized with ketamine–xylazine by intraperitoneal injection (100 mg/kg ketamine and 10 mg/kg xylazine), and were thereafter transcardially perfused with phosphate-buffered saline. Accordingly, most of the blood leukocytes in the brain microvessels were removed, and resident brain cells mostly contributed to the gene expression profile in hippocampus. Brains removed from the skull were snap-frozen immediately after dissection and were stored at −80 °C until use. Immediately before use, hippocampi were dissected as previously described [66], and were homogenized in TRIzol™ reagent using the TissueRuptor (Qiagen, Hilden, Germany).

### 4.3. Gene Expression Analysis

Pathway-focused qPCR arrays. RNA isolation from hippocampi was performed using the miRNeasy Tissue/Cells Advanced Micro Kit (Qiagen, Hilden, Germany) according to the manufacturer’s protocol, and RNA isolation from whole blood was performed using the modified PAXgene method [67]. RNA was quantified using a NanoDrop 2000 spectrophotometer (Thermo Scientific, Waltham, MA, USA). Reverse transcription of 400 ng total RNA was performed for each array experiment using the RT2 First Strand Kit (Qiagen, Hilden, Germany). The expression of 84 key genes involved in redox responses and of 84 genes related to inflammatory processes (Supplementary Table S1) was assessed by qPCR on 7500 Fast Real-Time PCR System (Applied Biosystems, Waltham, MA, USA) and was evaluated with RT^2^Profiler™ PCR Array Mouse Oxidative Stress (PAMM-065ZA, Qiagen, Hilden, Germany) and RT^2^ Profiler PCR Array Mouse NFkB Signaling Pathway (PAMM-025ZA, Qiagen, Hilden, Germany). The geometric mean of five housekeeping genes (*Actb*, *B2m*, *Gapdh*, *Gusb* and *Hsp90ab1*) was used to normalize the expression level of each transcript in hippocampus, while in blood four housekeeping genes (*Actb*, *B2m*, *Gapdh* and *Gusb*) were selected. The stability of the reference genes was established using the RefFinder algorithm (http://leonxie.esy.es/RefFinder/, accessed on 15 March 2022) [68]. The gene expression levels were calculated as 2^−∆CT^ values, and are reported in Supplementary Table S2. The fold change (FC) in gene expression was calculated as mean2^−ΔCT^ values in the interest mice group divided by the mean 2^−ΔCT^ values in the control group. The obtained results were presented as fold regulation (FR) values: if FC values were higher than 1, FR was equal to the FC value, and if FC values were lower than 1, FR was calculated as the negative inverse of FC.

Single gene qPCR. RNA isolation from whole blood was performed using the modified PAXgene method [67]. Reverse transcription of 500 ng total RNA was performed using the 90 High-Capacity cDNA Reverse Transcription Kit (Thermo Scientific, Waltham, MA, USA) according to the manufacturer’s protocol. The expression levels of *Gsr* and *Osgin1* were assessed by qPCR on 7500 Fast Real-Time PCR System (Applied Biosystems, Waltham, MA, USA). The primers are annotated in Supplementary Table S3. The gene expression levels were normalized against the geometric mean of two reference genes, *Gapdh* and *Tbp,* using the primers annotated in Supplementary Table S3. The stability of reference genes was assessed with the RefFinder algorithm (http://leonxie.esy.es/RefFinder/, accessed on 15 March 2022). The gene expression levels of *Gsr* and *Osgin1* are presented as 2^−ΔCT^values in Supplementary Table S2.

### 4.4. GEO Data Mining

The following gene expression datasets, reporting the gene expression levels in different brain regions from AD patients and controls were retrieved from the Gene Expression Omnibus database (GEO): GSE1297 (hippocampus), GSE48350 (hippocampus), GSE185909 (frontal cortex), GSE28146 (grey matter), GSE37264 (neocortex) and GSE118553 (entorhinal cortex). The GEO2R analysis tool was used to screen the differentially expressed genes between AD patients and controls. The adjusted *p*-value was calculated using the Benjamini and Hochberg procedure (the false discovery rate method).

### 4.5. Statistical Analysis

Statistical analysis was performed using the Statistical Package for Social Science (SPSS, version 17.0) and GraphPad Prism 8.4.3. Results are presented as mean ± standard error of the mean (SEM) or standard deviation (SD). The differences in gene expression between AT mice and the corresponding age-matched controls were evaluated using the non-parametric Mann–Whitney U-test. Only changes in gene expression with 1.50 > FR > 1.5 and *p* < 0.05 were considered significant. Correlations between continuous variables were performed using the Pearson test. Correlations with *p* < 0.05 and r < −0.8 or r > 0.8 were considered significant.

## 5. Conclusions

Altogether, the investigated double transgenic AT mouse model, which recapitulates human amyloid and TAU pathology in terms of neurologic, cognitive, behavioral and psychologic deficits, exhibits specific inflammation and redox gene expression disturbances in the brain and peripheral blood that partly overlap with those detected in AD patients. The identified genes might be valuable candidate biomarkers for preclinical drug development, which could be readily translated into clinical trials. In this context, we appreciate that AT mice represent an operative mouse model for testing new anti-inflammatory and antioxidant therapies in AD by following-up the expression changes in the identified genes.

## Figures and Tables

**Table 1:** Comparison of genes dysregulated in the hippocampus of AT mice (|FR| > 2, *p* < 0.05) and in post-mortem samples of AD brains from different microarray data sets (FR > |1.5| and nominal *p*-value < 0.05). HP = hippocampus; FCX = frontal cortex; GM = grey matter; NCX = neocortex; EC = entorhinal cortex; * adjusted *p*-value < 0.05.

Gene	AT vs. WT (FC > |2|)	GEODATASET Human Brain	Significance	Ref
***Il19***	↑FR = 3.66; *p* =0.034	↑7 AD vs. 4 CTRL (FCX)	FR = 1.80; *p* =0.03	[GSE185909]
***F2r***	↑FR = 2.84; *p* =0.004	↑7 incipient AD vs. 9 CTRL (HP)	FR = 2; *p* =0.004	[GSE1297]
		↑7 severe AD vs. 9 CTRL (HP)	FR = 2.03; *p* =0.002	[GSE1297]
***Tlr6***	↑FR = 2.75; *p* =0.001	↑ 7 AD vs. 4 CTRL (FCX)	FR = 1.62; *p* =0.024	[GSE185909]
***Il1b***	↑FR = 2.90; *p* =0.006	↓7 severe AD vs. 8 CTRL (GM)	FR = −10.86; *p* =0.00000698	[GSE28146]
***Lta***	↑FR = 2.17; *p* =0.034	↑7 AD vs. 4 CTRL (FCX)	FR = 1.61; *p* =0.024	[GSE185909]
		↑7 incipient AD vs. 8 CTRL (GM)	FR = 3.51; *p* =0.002	[GSE28146]
		↑8 AD vs. 8 CTRL (NCX)	FR = 1.57; *p* =0.05	[GSE37264]
***Csf2***	↓FR = −2.20; *p* =0.021	↑7 incipient AD vs. 9 CTRL (HP)	FR = 1.98; *p* =0.02	[GSE1297]
		↑7 severe AD vs. 9 CTRL (HP)	FR = 2.41; *p* =0.00009546	[GSE1297]
***Gpx3***	↑FR = 2.36; *p* =0.016	↑ 36 AD vs. 16 CTRL (EC)	FR = 1.66; *p* =0.000025 *	[GSE118553]
***Nox1***	↑FR = 2.14; *p* =0.004	↑ 7 incipient AD vs. 9 CTRL (HP)	FR = 1.66; *p* =0.04	[GSE1297]

**Table 2:** Gene expression changes in the blood of AT mice and mild AD patients [24]. Results are expressed as FR values. Only the genes with FR > 1.8 (*p* < 0.05) in mice are represented. Comparisons between diseased individuals (patients or mice) and the corresponding age-matched controls were made using the Mann–Whitney U-test; differences were considered significant for *p* < 0.05.

Mice Blood (9 AT vs. 7 WT)			Human Blood (38 AD vs. 38 CTRL)		
Gene	FR	*p*-Value	Gene	FR	*p*-Value
*Ltbr*	2.03	0.001	*LTBR*	1.54	0.001
*F2r*	1.84	0.012	*F2R*	1.53	0.006
*Il1b*	3.30	<0.001	*IL1B*	1.79	<0.001
*Csf1*	2.13	0.016	*CSF1*	1.76	<0.001
*Gsr*	2.23	<0.001	*GSR*	3.93	<0.001

**Table 3:** Expression changes in selected redox genes in the blood of AT mice with various ages and of mild AD patients [24]. Results are expressed as FR values. Comparisons between diseased individuals (patients or mice) and the corresponding age-matched controls were made using the Mann–Whitney U-test, and differences were considered significant for *p* < 0.05.

	Mice Blood Data				Human Blood Data [24]		
	21 AT vs. 12 WT		7 AT vs. 8 WT			38 MCI vs. 38 CTRL	
	48.5 ± 4.9 weeks		37.1 ± 0.8 weeks				
**Gene**	**FR**	***p*** **-value**	**FR**	***p*** **-value**	**Gene**	**FR**	***p*** **-value**
*Gsr*	2.08	<0.001	1.70	0.001	*GSR*	3.93	<0.001
*Osgin1*	2.72	<0.001	1.69	0.001	*OSGIN1*	-	-

## Data Availability

Data are contained within the article or Supplementary Materials.

## Supplementary Materials

The following supporting information can be downloaded at: https://www.mdpi.com/article/10.3390/ijms23105799/s1.
