# Supplementary material for: Altered Blood and Brain Expression of Inflammation and Redox Genes in Alzheimer’s Disease, Common to APPV717I × TAUP301L Mice and Patients
Source: Int J Mol Sci. 2022 May 21;23(10):5799. doi: 10.3390/ijms23105799 (PMC9144576; doi:10.3390/ijms23105799)
Supplement: Supplementary file 1 [file ijms-23-05799-s001.zip › Supplementary Table 1.pdf]

Supplementary Table 1. List of the analyzed transcripts using qPCR array.

A. RT² Profiler™ PCR Array Mouse NFkB Signaling Pathway, Qiagen

NFκB Signaling

NFκB Signaling Ligands & Receptors: Card10, Ccl2 (MCP-1), Cd27, Cd40, Csf2 (GMCSF), Egfr, F2r (Par1), Fasl, Ifng, Il10, Il1a, Il1b, Il1r1, Lta (Tnfb), Ltbr, Nod1, Tlr1, Tlr2, Tlr3, Tlr4, Tlr6, Tlr9, Tnf, Tnfrsf10b (Dr5), Tnfrsf1a (Tnfr1), Tnfrsf1b, Tnfsf10 (Trail), Tnfsf14.

Signaling Downstream of NFκB: Fadd, Irak1, Irak2, Irf1, Map3k1 (Mekk1), Mapk3 (Erk1), Myd88, Ripk1, Ripk2, Tnfaip3, Tollip, Tradd, Traf2, Traf3, Traf5, Traf6.

Cytoplasmic Sequestering / Releasing of NFκB: Bcl3, Chuk (IKBKA), Ikbkb (IKKbeta), Ikbke, Ikbkg, Nfkbia (Iκbα, Mad3).

Transcription Factors: Nfkb1, Nfkb2, Rel, Rela, Relb.

NFκB Responsive Genes

Immune Response: Ccl2 (MCP-1), Ccl5 (Rantes), Csf1 (Mcsf), Csf2 (GMCSF), Csf3 (Gcsf), Icam1, Ifng, Irf1, Lta (Tnfb), Stat1, Tnf.

Apoptosis: Agt, Bcl2a1a (Bfl-1, A1), Bcl2l1 (Bcl-XL), Birc3 (cIAP1, cIAP2).

Other NFκB Signaling Genes

Kinases: Akt1, Eif2ak2, Raf1, Tbk1, Zap70.

Transcription Factors: Atf1, Atf2 (Creb2), Crebbp (CBP), Egr1, Elk1, Fos, Jun, Smad3.

Other NFκB Signaling Genes: Bcl10, Card11, Casp1 (Ice), Casp8 (Flice), Cflar (Casper), Hmox1, Slc20a1.

B. RT² Profiler™ PCR Array Mouse Oxidative Stress and Antioxidant Defense, Qiagen

Antioxidants

Glutathione Peroxidases (GPx): Gpx1, Gpx2, Gpx3, Gpx4, Gpx5, Gpx6, Gpx7, Gstk1, Gstp1.

Peroxiredoxins: (TPx)Ehd2,Prdx1,Prdx2,Prdx3,Prdx4,Prdx5,Prdx6 (Aop2).

Other Peroxidases: Apc, Cat, Ctsb, Duox1, Epx, Lpo, Mpo, Ptgs1 (Cox1), Ptgs2 (Cox2), Rag2, Serpinb1b, Tpo.

Other Antioxidants: Alb, Gsr, Sod1, Sod3, Srxn1, Txnrd1, Txnrd2, Txnrd3.

Reactive Oxygen Species (ROS) Metabolism

Superoxide Dismutases (SOD): Sod1, Sod2, Sod3.

Other Superoxide Metabolism Genes: Ccs, Cyba, Ncf1, Ncf2, Nos2 (iNos), Nox1, Nox4, Noxa1, Noxo1, Recql4, Scd1, Ucp2.

Other Reactive Oxygen Species (ROS) Metabolism Genes: Aox1, Fmo2, Il19, Il22.

Oxidative Stress Responsive Genes: Als2, Apoe, Cat, Ccl5 (Rantes), Ctsb, Duox1, Epx, Ercc2 (Xpd), Ercc6, Fth1, Gclc, Gclm, Gpx1, Gpx2, Gpx3, Gpx4, Gpx5, Gpx6, Gpx7, Gsr, Gss, Hmox1, Hspa1a (hsp70A1), Idh1, Krt1, Mpo, Nqo1, Park7, Prdx1, Prdx2, Prdx6 (Aop2), Prnp, Psmb5, Sod1, Sqstm1, Tpo, Txn1, Txnip, Txnrd1, Txnrd2, Ucp3, Xpa.

Oxygen Transporters

Atr, Cygb, Dnm2, Fancc, Ift172, Mb, Ngb, Slc38a1, Vim.
