# Supplementary material for: Altered Blood and Brain Expression of Inflammation and Redox Genes in Alzheimer’s Disease, Common to APPV717I × TAUP301L Mice and Patients
Source: Int J Mol Sci. 2022 May 21;23(10):5799. doi: 10.3390/ijms23105799 (PMC9144576; doi:10.3390/ijms23105799)
Supplement: Supplementary file 1 [file ijms-23-05799-s001.zip › Supplementary Table 2.pdf]

Supplementary Table 2. Gene expression levels of investigated transcripts. Data is presen

					2^-ΔCT	2^-ΔCT	2^-ΔCT
**CODE**	**Hippocampus/Bl**	**Genotype**	**Sex**	**AGE_week**	**Gsr**	**Osgin1**	**Agt**
AT_452	Hippocampus	AT_transge	Male	54.00			0.057239
AT_458	Hippocampus	AT_transge	Male	51.00			0.228420
AT_459	Hippocampus	AT_transge	Male	51.00			0.132632
AT_462	Hippocampus	AT_transge	Male	53.00			0.025606
AT_471	Hippocampus	AT_transge	Male	51.00			0.114265
AT_563	Hippocampus	AT_transge	Male	60.00			0.072441
AT_574	Hippocampus	AT_transge	Female	58.00			0.118317
AT_576	Hippocampus	AT_transge	Male	58.00			0.087665
AT_579	Hippocampus	AT_transge	Male	57.00			0.037482
AT_580	Hippocampus	AT_transge	Male	57.00			0.146782
WT_146	Hippocampus	Wild type	Male	54.00			0.111290
WT_154	Hippocampus	Wild type	Male	51.00			0.028307
WT_155	Hippocampus	Wild type	Male	51.00			0.072076
WT_156	Hippocampus	Wild type	Male	51.00			0.090257
WT_157	Hippocampus	Wild type	Female	52.00			0.179284
WT_160	Hippocampus	Wild type	Male	52.00			0.025007
WT_206	Hippocampus	Wild type	Female	59.00			0.011143
WT_207	Hippocampus	Wild type	Female	59.00			0.020458
AT_452	Blood	AT_transge	Male	54.00			0.001010
AT_458	Blood	AT_transge	Male	51.00			0.000299
AT_462	Blood	AT_transge	Male	53.00			0.000021
AT_471	Blood	AT_transge	Male	51.00			0.000214
AT_563	Blood	AT_transge	Male	60.00			0.000230
AT_574	Blood	AT_transge	Female	58.00			0.000019
AT_576	Blood	AT_transge	Male	58.00			0.000180
AT_579	Blood	AT_transge	Male	57.00			0.000000
AT_580	Blood	AT_transge	Male	57.00			0.000013
WT_154	Blood	Wild type	Male	51.00			0.000100
WT_155	Blood	Wild type	Male	51.00			0.000800
WT_156	Blood	Wild type	Male	51.00			0.005100
WT_158	Blood	Wild type	Female	52.00			0.000000
WT_160	Blood	Wild type	Female	52.00			0.000053
WT_206	Blood	Wild type	Female	59.00			0.000202
WT_207	Blood	Wild type	Female	59.00			0.000000
AT 537	Blood	AT_transge	Female	52.00	2.99771	0.06752	
AT 538	Blood	AT_transge	Female	52.00	3.99819	0.08340	
AT 540	Blood	AT_transge	Male	52.00	4.46170	0.18295	
AT 544	Blood	AT_transge	Male	51.00	3.26477	0.09451	
AT 547	Blood	AT_transge	Male	48.00	2.98707	0.10393	
AT 548	Blood	AT_transge	Male	48.00	3.41919	0.07760	

AT 549	Blood	AT_transge	Male	48.00	2.31873	0.07424
AT 552	Blood	AT_transge	Female	49.00	2.02624	0.08724
AT 553	Blood	AT_transge	Female	49.00	2.03449	0.06244
AT 554	Blood	AT_transge	Female	49.00	2.44676	0.08335
AT 590	Blood	AT_transge	Female	52.00	2.58184	0.11756
AT 591	Blood	AT_transge	Female	52.00	2.61705	0.13825
AT 597	Blood	AT_transge	Male	52.00	1.78480	0.11955
AT 605	Blood	AT_transge	Female	51.00	2.86297	0.14703
AT 607	Blood	AT_transge	Male	50.00	2.33519	0.13559
AT 624	Blood	AT_transge	Female	44.00	1.89346	0.14537
AT 632	Blood	AT_transge	Male	44.00	1.48789	0.06742
AT 636	Blood	AT_transge	Female	43.00	2.17075	0.07401
AT 643	Blood	AT_transge	Male	44.00	1.78526	0.12775
AT 645	Blood	AT_transge	Female	39.00	2.34430	0.16616
AT 646	Blood	AT_transge	Male	39.00	2.14577	0.12136
WT 197	Blood	Wild type	Male	57.00	1.01563	0.02027
WT 198	Blood	Wild type	Male	57.00	1.04598	0.02596
WT 199	Blood	Wild type	Male	57.00	1.16306	0.02692
WT 203	Blood	Wild type	Male	49.00	1.31182	0.03496
WT 204	Blood	Wild type	Male	49.00	0.94014	0.03890
WT 208	Blood	Wild type	Male	41.00	1.30158	0.03608
WT 210	Blood	Wild type	Male	54.00	1.32751	0.03692
WT 211	Blood	Wild type	Male	54.00	0.99250	0.01866
WT 215	Blood	Wild type	Female	45.00	1.51500	0.06505
WT 216	Blood	Wild type	Female	45.00	1.92478	0.08915
WT 219	Blood	Wild type	Male	43.00	0.73703	0.03192
WT 220	Blood	Wild type	Female	43.00	1.58190	0.05385
AT 716	Blood	AT_transge	Female	38.00	1.48716	0.11127
AT 718	Blood	AT_transge	Female	38.00	1.42125	0.10827
AT 720	Blood	AT_transge	Female	38.00	1.34626	0.07365
AT 724	Blood	AT_transge	Male	38.00	2.82202	0.09250
AT 725	Blood	AT_transge	Male	38.00	1.64831	0.06373
AT 733	Blood	AT_transge	Female	36.00	1.26580	0.08279
AT 735	Blood	AT_transge	Female	36.00	1.41470	0.06083
WT 283	Blood	Wild type	Female	37.00	1.03376	0.05300
WT 284	Blood	Wild type	Female	37.00	1.32198	0.04993
WT 287	Blood	Wild type	Female	37.00	1.14291	0.04763
WT 288	Blood	Wild type	Female	36.00	0.52029	0.02530
WT 289	Blood	Wild type	Female	36.00	1.27542	0.04541
WT 292	Blood	Wild type	Male	37.00	0.42435	0.04927
WT 293	Blood	Wild type	Male	37.00	0.91196	0.07313
WT 294	Blood	Wild type	Male	37.00	1.04303	0.05837

ted as $2^{-\Delta CT}$ values.

$2^{-\Delta CT}$	$2^{-\Delta CT}$	$2^{-\Delta CT}$	$2^{-\Delta CT}$	$2^{-\Delta CT}$	$2^{-\Delta CT}$	$2^{-\Delta CT}$	$2^{-\Delta CT}$	$2^{-\Delta CT}$
**Akt1**	**Atf1**	**Atf2**	**Bcl10**	**Bcl2a1a**	**Bcl2l1**	**Bcl3**	**Birc3**	**Card10**
0.195216	0.069019	0.175938	0.044599	0.001681	0.153163	0.000141	0.003408	0.002987
0.226842	0.076402	0.219115	4.227371	0.002524	0.230009	0.000459	0.003194	0.005718
0.254458	0.078319	0.187570	0.054617	0.001961	0.216960	0.000266	0.003185	0.002735
0.183344	0.064822	0.184619	0.045205	0.001877	0.155246	0.000187	0.002426	0.002986
0.230119	0.082495	0.250079	0.054805	0.002542	0.184342	0.000305	0.002422	0.003425
0.100339	0.070460	0.219601	0.029217	0.004591	0.163001	0.000183	0.003201	0.003807
0.106633	0.100184	0.236633	0.034933	0.003379	0.180582	0.000109	0.003498	0.003379
0.121426	0.129242	0.236211	0.035113	0.004904	0.179014	0.000187	0.002628	0.003956
0.093581	0.062602	0.175843	0.028803	0.004589	0.138923	0.000203	0.002082	0.003359
0.109709	0.092254	0.250303	0.033072	0.005343	0.161740	0.000210	0.003674	0.003726
0.183315	0.060892	0.188469	0.040174	0.003049	0.162939	0.000241	0.002494	0.002767
0.190429	0.064584	0.215733	0.048272	0.001936	0.177676	0.000108	0.003080	0.002351
0.212517	0.088122	0.268995	0.055386	0.001792	0.194205	0.000312	0.001881	0.004203
0.190807	0.074335	0.196172	0.053667	0.001228	0.188180	0.000129	0.001018	0.004045
0.243218	0.065169	0.193488	0.051130	0.002525	0.211733	0.000148	0.002065	0.003285
0.123150	0.057451	0.186660	0.034879	0.011747	0.151615	0.000140	0.002470	0.003236
0.034489	0.012712	0.037222	0.006356	0.011066	0.037481	0.000069	0.000262	0.002025
0.145470	0.058266	0.148527	0.025538	0.009544	0.153765	0.000078	0.001479	0.003041
0.070260	0.107980	0.069774	0.168269	0.021180	0.663809	0.002818	0.029336	0.004271
0.109653	0.096122	0.058355	0.201802	0.016076	1.165550	0.005843	0.056760	0.005230
0.048490	0.072485	0.044312	0.122753	0.013451	0.511863	0.001444	0.026166	0.002808
0.095493	0.061279	0.036945	0.188357	0.012617	1.730924	0.004523	0.027423	0.006715
0.071606	0.065435	0.041701	0.141240	0.015476	0.798972	0.005285	0.024117	0.001518
0.118838	0.085797	0.044720	0.156807	0.029300	0.542264	0.011655	0.038395	0.007583
0.090501	0.051620	0.037268	0.186091	0.020250	0.765291	0.006147	0.024931	0.008573
0.092727	0.093372	0.048669	0.162571	0.015401	1.013353	0.005187	0.029547	0.005716
0.062855	0.065980	0.043833	0.114878	0.011583	0.822550	0.004359	0.031866	0.006887
0.059210	0.041580	0.028010	0.118410	0.043950	0.532890	0.004990	0.048760	0.002100
0.055630	0.036450	0.028600	0.171000	0.007150	1.103510	0.002640	0.028600	0.001390
0.045200	0.051560	0.056430	0.123490	0.017490	0.718210	0.005500	0.025080	0.003650
0.055460	0.044737	0.036337	0.164665	0.009736	1.655884	0.004297	0.024478	0.003466
0.072631	0.060235	0.056985	0.129116	0.015375	0.597387	0.011100	0.053169	0.000151
0.043489	0.062362	0.037338	0.085188	0.026402	0.640292	0.002834	0.021445	0.005990
0.040340	0.041763	0.027553	0.073729	0.061145	0.341125	0.003492	0.021920	0.001871

2^-ΔCT	2^-ΔCT	2^-ΔCT	2^-ΔCT	2^-ΔCT	2^-ΔCT	2^-ΔCT	2^-ΔCT	2^-ΔCT
**Card11**	**Casp1**	**Casp8**	**Ccl2**	**CCL5**	**Cd27**	**Cd40**	**Cflar**	**Chuk**
0.000318	0.004853	0.008276	0.000463	0.0055960	0.004528	0.001443	0.057637	0.121005
0.000549	0.005798	0.007704	0.000538	0.0040510	0.004455	0.001564	0.055160	0.154938
0.000467	0.004861	0.008464	0.000385	0.0039400	0.004059	0.001008	0.061448	0.159929
0.000327	0.006581	0.006957	0.000992	0.0048460	0.003833	0.001504	0.063049	0.144849
0.000378	0.004679	0.008792	0.000389	0.0034755	0.004844	0.001307	0.067473	0.149732
0.001109	0.003834	0.008390	0.001213	0.0081305	0.001634	0.002083	0.042187	0.252255
0.001512	0.004306	0.009231	0.000672	0.0079495	0.001391	0.000998	0.050791	0.269943
0.001351	0.005404	0.010295	0.000690	0.0092850	0.002062	0.001252	0.070226	0.280903
0.001020	0.003267	0.007053	0.000687	0.0084440	0.001890	0.000721	0.046467	0.180786
0.001264	0.002804	0.010910	0.001799	0.0097025	0.002170	0.001392	0.052620	0.246857
0.000305	0.003134	0.004918	0.000752	0.0042035	0.002691	0.000857	0.060054	0.150977
0.000093	0.005591	0.005514	0.000112	0.0024460	0.003348	0.001052	0.042315	0.175230
0.000328	0.015257	0.007576	0.000780	0.0032530	0.003390	0.000836	0.062312	0.187590
0.000338	0.005374	0.007978	0.000585	0.0027770	0.003002	0.000821	0.048704	0.163821
0.000610	0.004276	0.005720	0.000482	0.0042945	0.003378	0.000931	0.048709	0.162704
0.000838	0.004040	0.006563	0.000832	0.0052700	0.002077	0.000724	0.042349	0.174160
0.000543	0.000778	0.001403	0.000201	0.0027555	0.000574	0.000299	0.008926	0.041015
0.000509	0.005332	0.004706	0.000782	0.0041180	0.001872	0.000671	0.038978	0.180341
0.012420	0.090800	0.054744	0.001834	0.1790670	0.068814	0.009813	0.134795	0.140519
0.044533	0.110415	0.053698	0.000593	0.1918630	0.100204	0.021065	0.150832	0.083101
0.010775	0.056478	0.036495	0.000000	0.1486560	0.056088	0.014719	0.193960	0.127082
0.021665	0.082557	0.043936	0.000023	0.1676930	0.083132	0.009831	0.117566	0.042439
0.023135	0.059384	0.027322	0.001088	0.1850385	0.069648	0.017412	0.139295	0.082825
0.015593	0.054677	0.039475	0.002484	0.2014560	0.070173	0.007427	0.112427	0.072648
0.011631	0.061814	0.020820	0.000420	0.2378880	0.037268	0.002888	0.103959	0.060963
0.005716	0.052890	0.027378	0.000705	0.1946345	0.076369	0.011274	0.148562	0.091451
0.019616	0.054719	0.022377	0.001305	0.1645955	0.068782	0.020449	0.114878	0.073210
0.029810	0.093551	0.057587	0.001281	0.2018340	0.033537	0.031291	0.078667	0.072892
0.041290	0.066620	0.043649	#NULL!	0.1278365	0.051908	0.024216	0.131406	0.050489
0.044580	0.077615	0.021680	0.000668	0.2388695	0.035959	0.015981	0.109764	0.092942
0.028313	0.062396	0.025517	0.000000	0.1433795	0.039764	0.014255	0.149436	0.060690
0.086975	0.133669	0.058995	0.002589	0.1774905	0.050301	0.036065	0.130918	0.074673
0.022668	0.045651	0.028892	0.000404	0.2636090	0.032731	0.014546	0.113976	0.090046
0.011346	0.062864	0.030999	0.000898	0.3168210	0.029124	0.016270	0.106459	0.110980

2^-ΔCT	2^-ΔCT	2^-ΔCT	2^-ΔCT	2^-ΔCT	2^-ΔCT	2^-ΔCT	2^-ΔCT	2^-ΔCT
**Crebbp**	**Csf1**	**Csf2**	**Csf3**	**Egfr**	**Egr1**	**Eif2ak2**	**Elk1**	**F2r**
0.147946	0.037764	0.024233	0.000287	0.014610	0.192528	0.010260	0.154229	0.015768
0.135819	0.038734	0.050059	0.000425	0.017821	0.216098	0.017214	0.104369	0.043277
0.142152	0.035538	0.024783	0.000415	0.016579	0.240732	0.009262	0.166720	0.015256
0.185903	0.036212	0.022602	0.000273	0.012197	0.177099	0.010115	0.187196	0.008388
0.178062	0.028566	0.022104	0.000294	0.014482	0.129449	0.012608	0.167294	0.017463
0.147927	0.018363	0.078181	0.001413	0.008049	0.139947	0.012031	0.132398	0.033100
0.176866	0.013515	0.044834	0.001054	0.007094	0.173226	0.011365	0.134971	0.027984
0.214366	0.018050	0.068306	0.001351	0.010224	0.139482	0.012852	0.169358	0.038424
0.129620	0.012537	0.070921	0.001155	0.005495	0.123481	0.009178	0.111287	0.014806
0.163998	0.016308	0.060027	0.001383	0.009174	0.084304	0.012974	0.075454	0.055236
0.140866	0.030236	0.098236	0.000397	0.014105	0.197839	0.010764	0.124343	0.014910
0.182671	0.030339	0.053191	0.000304	0.014961	0.443597	0.009870	0.125636	0.008834
0.198286	0.034810	0.076185	0.000366	0.022966	0.474886	0.017285	0.151318	0.011806
0.163821	0.040672	0.041527	0.000228	0.015095	0.267978	0.012176	0.152850	0.016291
0.143619	0.028170	0.062514	0.000414	0.016180	0.216182	0.008203	0.160464	0.011362
0.134762	0.023658	0.176592	0.000729	0.008600	0.277102	0.009153	0.148495	0.005674
0.030869	0.006489	0.160684	0.000586	0.002408	0.038004	0.002171	0.039344	0.001622
0.143468	0.021624	0.151648	0.000892	0.008782	0.210049	0.008308	0.150600	0.005520
0.020888	0.010812	0.000129	0.000000	0.001160	0.000000	0.015612	0.004706	0.025895
0.024878	0.016187	0.000024	0.000475	0.000028	0.000730	0.021212	0.010034	0.018466
0.007109	0.004958	0.000098	0.000000	0.000536	0.001173	0.009779	0.009124	0.012993
0.045485	0.015214	0.002198	0.000000	0.000000	0.003243	0.019391	0.004555	0.021665
0.014340	0.011020	0.005166	0.000000	0.000000	0.001416	0.007846	0.003560	0.013852
0.018162	0.015486	0.000015	0.000000	0.000000	0.001616	0.010217	0.005787	0.024983
0.022314	0.008754	0.002732	0.000000	0.000000	0.000660	0.010852	0.006408	0.034294
0.014570	0.010966	0.007185	0.000000	0.000000	0.005045	0.010302	0.006042	0.033242
0.014460	0.011266	0.005184	0.000088	0.000000	0.000350	0.008421	0.005148	0.019481
0.025241	0.003453	0.024129	0.000295	0.000000	0.001212	0.030436	0.004340	0.005161
0.072398	0.012024	0.000166	#NULL!	0.000000	0.009369	0.013908	0.005807	0.009113
0.024222	0.000834	0.010691	0.000035	0.000283	0.000088	0.019404	0.008564	0.006908
0.065046	0.007127	0.008714	0.000000	0.000000	0.006604	0.025341	0.003212	0.019608
0.039466	0.008297	0.000349	0.000000	0.000000	0.004477	0.017908	0.002974	0.015917
0.012064	0.005143	0.044096	0.000028	0.000000	0.002536	0.016140	0.004865	0.017299
0.011665	0.001269	0.014066	0.000026	0.000000	0.000102	0.017804	0.003327	0.012503

2^-ΔCT	2^-ΔCT	2^-ΔCT	2^-ΔCT	2^-ΔCT	2^-ΔCT	2^-ΔCT	2^-ΔCT	2^-ΔCT
**Fadd**	**Fasl**	**Fos**	**Hmox1**	**Icam1**	**Ifng**	**Ikbkb**	**Ikbke**	**Ikbkg**
0.011150	0.000528	0.025793	0.004655	0.006186	0.000118	0.052307	0.001623	0.048804
0.013600	0.001490	0.030390	0.004157	0.007493	0.000030	0.048690	0.003129	0.043882
0.012221	0.000595	0.043752	0.004696	0.005741	0.000032	0.049566	0.001649	0.046893
0.009838	0.000314	0.011301	0.004080	0.004719	0.000026	0.047125	0.001163	0.042178
0.011284	0.000553	0.006572	0.004878	0.005156	0.000028	0.045767	0.001689	0.046729
0.014013	0.001291	0.013630	0.006448	0.003408	0.000055	0.023083	0.000823	0.048461
0.012523	0.001713	0.010531	0.004879	0.002901	0.000103	0.023047	0.001203	0.052582
0.012414	0.001332	0.016268	0.005954	0.003983	0.000023	0.024318	0.000885	0.045378
0.010914	0.001131	0.006860	0.006269	0.003049	0.000025	0.018103	0.000472	0.055259
0.013246	0.001876	0.014296	0.005343	0.003831	0.000012	0.024891	0.000736	0.046771
0.013531	0.000632	0.018872	0.005022	0.004311	0.000038	0.038007	0.001452	0.042464
0.010008	0.000377	0.031409	0.004356	0.003663	0.000000	0.044728	0.001745	0.042023
0.009009	0.001199	0.056549	0.003710	0.005702	0.000040	0.052036	0.001842	0.039163
0.007653	0.000833	0.047045	0.004744	0.004979	0.000097	0.052200	0.001620	0.037168
0.009960	0.000672	0.029367	0.004551	0.004217	0.000054	0.040116	0.002153	0.044204
0.009027	0.000393	0.027176	0.014973	0.002487	0.000047	0.028726	0.001305	0.038167
0.006955	0.000210	0.005056	0.008804	0.001171	0.000044	0.017485	0.000566	0.009701
0.007806	0.000258	0.010018	0.012164	0.001607	0.000228	0.031880	0.000996	0.032102
0.016733	0.008251	0.057465	0.003445	0.010963	0.002063	0.017934	0.022233	0.010372
0.011606	0.012967	0.102309	0.003474	0.018854	0.001901	0.049071	0.026296	0.010533
0.011078	0.007060	0.015996	0.002808	0.011469	0.002847	0.012377	0.009124	0.008395
0.008210	0.003005	0.064326	0.014901	0.021665	0.001621	0.065677	0.017720	0.004339
0.011729	0.009931	0.013105	0.004061	0.018026	0.002736	0.019186	0.014241	0.004475
0.013669	0.004934	0.026043	0.006377	0.015702	0.001650	0.031842	0.016143	0.005747
0.009253	0.010852	0.017751	0.005735	0.013360	0.001879	0.024418	0.010927	0.003434
0.009952	0.012167	0.018442	0.005010	0.019629	#NULL!	0.019092	0.012337	0.007336
0.011992	0.010154	0.014866	0.002683	0.013775	0.004836	0.018302	0.013967	0.008191
0.016423	0.006533	0.022435	0.005532	0.033770	0.001929	0.056013	0.015430	0.007556
0.015977	0.003701	0.024216	0.006399	0.019806	0.000965	0.058806	0.013250	0.003359
0.009837	0.010617	0.004282	0.005308	0.021381	0.002904	0.031521	0.007826	0.007004
0.014554	0.004638	0.032523	0.002955	0.014454	0.002005	0.037359	0.014156	0.005911
0.012402	0.004385	0.032730	0.002893	0.029909	0.001216	0.050651	0.016139	0.008070
0.010797	0.016140	0.001744	0.006881	0.016479	0.003817	0.025152	0.007478	0.002589
0.014263	0.013682	0.004608	0.003666	0.013307	0.002062	0.024491	0.008364	0.003795

2^-ΔCT	2^-ΔCT	2^-ΔCT	2^-ΔCT	2^-ΔCT	2^-ΔCT	2^-ΔCT	2^-ΔCT	2^-ΔCT
**Il10**	**Il1a**	**Il1b**	**Il1r1**	**Irak1**	**Irak2**	**Irf1**	**Jun**	**Lta**
0.000027	0.002478	0.000611	0.024402	0.053406	0.036986	0.021540	0.197941	0.000241
0.000030	0.002649	0.001004	0.014678	0.063362	0.056711	0.017821	0.139638	0.000505
0.000041	0.004599	0.001330	0.039706	0.071570	0.041392	0.020985	0.206684	0.000118
0.000036	0.003383	0.000489	0.040741	0.065727	0.036973	0.024057	0.262907	0.000344
0.000028	0.002614	0.001298	0.018978	0.053306	0.029574	0.021204	0.155012	0.000279
0.000532	0.004989	0.001494	0.016435	0.051224	0.030458	0.009245	0.168750	0.000846
0.000307	0.004397	0.002153	0.015100	0.055581	0.025220	0.008794	0.144659	0.000799
0.000609	0.004870	0.001573	0.029732	0.060713	0.031646	0.011826	0.232959	0.000765
0.000469	0.003967	0.001970	0.020226	0.045827	0.022598	0.009436	0.167514	0.000945
0.000531	0.006398	0.002155	0.009563	0.044248	0.024378	0.006137	0.119224	0.000645
0.000059	0.003178	0.000438	0.015436	0.052644	0.043057	0.016543	0.126079	0.000191
0.000005	0.001733	0.000512	0.013298	0.057804	0.040874	0.018806	0.114810	0.000093
0.000037	0.002931	0.000738	0.016929	0.062312	0.041684	0.021280	0.126364	0.000212
0.000052	0.001953	0.000368	0.026464	0.051126	0.039287	0.024184	0.213187	0.000122
0.000035	0.002439	0.000358	0.016292	0.054800	0.043294	0.018203	0.132156	0.000145
0.000247	0.003795	0.001201	0.017806	0.048311	0.032095	0.019894	0.144435	0.000458
0.000223	0.001656	0.000817	0.015868	0.021378	0.014909	0.007403	0.112057	0.000397
0.000137	0.003493	0.000719	0.020743	0.045085	0.031660	0.020037	0.145470	0.000407
0.000000	0.007134	1.070911	0.004097	0.030795	0.014071	0.107980	0.010590	0.000597
0.000000	0.004103	0.973337	0.001107	0.030628	0.025577	0.134998	0.010460	0.002423
0.000000	0.002158	0.537311	0.001474	0.020529	0.004406	0.045557	0.011548	0.000829
0.000000	0.001714	0.679028	0.000863	0.035440	0.027806	0.149845	0.012272	0.000751
0.000000	0.002132	0.437155	0.002994	0.026391	0.012924	0.079451	0.007023	0.002932
0.000000	0.001909	1.054871	0.002104	0.037087	0.015379	0.131859	0.011336	0.002590
0.000000	0.002329	0.362005	0.001102	0.024931	0.008224	0.061387	0.004691	#NULL!
0.000000	0.002007	0.945492	0.000394	0.042663	0.010161	0.093372	0.009679	0.001419
0.000000	0.002179	0.443860	0.001176	0.022690	0.010084	0.058241	0.006294	0.000605
0.000701	0.002025	0.301849	0.003525	0.025593	0.025771	0.113589	0.003911	0.000000
0.000000	0.000894	0.264640	0.002617	0.031296	0.023554	0.153053	0.006811	0.001155
0.000059	0.003091	0.182059	0.003178	0.018613	0.020796	0.078698	0.009769	0.000664
0.000000	0.001082	0.201325	0.002351	0.045677	0.020441	0.105667	0.008775	0.001595
0.000000	0.001436	0.357679	0.001922	0.023630	0.037079	0.156772	0.010722	0.001922
0.000000	0.001922	0.102721	0.003210	0.022356	0.009142	0.051360	0.004355	0.001708
0.002556	0.000989	0.122289	0.004576	0.024153	0.010960	0.073220	0.007332	0.001136

2^-ΔCT	2^-ΔCT	2^-ΔCT	2^-ΔCT	2^-ΔCT	2^-ΔCT	2^-ΔCT	2^-ΔCT	2^-ΔCT
**Ltbr**	**Map3k1**	**Mapk3**	**Myd88**	**Nfkb1**	**Nfkb2**	**Nfkbia**	**Nod1**	**Raf1**
0.014113	0.007994	0.374527	0.000532	0.039095	0.002172	0.084973	0.004623	0.172318
0.015514	0.013413	0.279275	0.000658	0.044494	0.002506	0.122407	0.008667	0.214606
0.011403	0.012565	0.322082	0.000967	0.034090	0.003229	0.158824	0.008290	0.191511
0.012540	0.008159	0.319220	0.000466	0.041310	0.002460	0.067575	0.004919	0.196503
0.013419	0.009621	0.228530	0.000677	0.043298	0.001981	0.080239	0.006260	0.166138
0.014915	0.011226	0.183386	0.001291	0.029217	0.003703	0.096921	0.013443	0.132398
0.012013	0.009690	0.190878	0.000833	0.030411	0.003775	0.155041	0.009825	0.125063
0.013398	0.009343	0.171722	0.001418	0.026982	0.003019	0.133800	0.014661	0.149493
0.010616	0.006445	0.169853	0.000875	0.024220	0.002359	0.132343	0.013252	0.101697
0.016422	0.013905	0.187083	0.001155	0.037467	0.003624	0.137905	0.015429	0.139830
0.014204	0.007301	0.261050	0.000692	0.038537	0.003049	0.086713	0.005849	0.159585
0.013862	0.009939	0.263764	0.000634	0.037612	0.003017	0.078418	0.004935	0.178912
0.017526	0.013561	0.313308	0.000848	0.039163	0.003120	0.091230	0.004232	0.169065
0.015095	0.013510	0.309967	0.001178	0.042107	0.003308	0.085121	0.008610	0.196172
0.013051	0.007869	0.236567	0.000827	0.039290	0.002356	0.085983	0.005374	0.185606
0.011347	0.006608	0.221978	0.000729	0.029946	0.002721	0.074248	0.008249	0.137594
0.008101	0.005419	0.054496	0.000531	0.010469	0.001255	0.048104	0.005610	0.079236
0.010158	0.006210	0.239616	0.000867	0.027561	0.002211	0.072735	0.006890	0.135729
0.026623	0.016275	0.048659	0.001531	0.057069	0.008844	0.048323	0.005916	0.072737
0.025934	0.027604	0.053698	0.001774	0.092848	0.009106	0.052959	0.019519	0.087233
0.021401	0.007208	0.041344	0.001164	0.039936	0.003505	0.025627	0.002173	0.056871
0.038782	0.028987	0.048079	0.002984	0.085469	0.009301	0.041855	0.009109	0.127763
0.026760	0.009527	0.045633	0.000638	0.056965	0.004698	0.052418	0.008067	0.055407
0.047930	0.016143	0.066388	0.007479	0.056998	0.004701	0.063244	0.008651	0.101325
0.038051	0.007110	0.073002	0.001434	0.051620	0.002428	0.028244	0.005241	0.077701
0.036125	0.007387	0.058685	0.001553	0.050037	0.004610	0.059504	0.004453	0.103603
0.020028	0.006887	0.047635	0.001332	0.061990	0.006516	0.047635	0.007749	0.069260
0.017972	0.037731	0.039882	0.001345	0.105983	0.011453	0.062582	0.017725	0.069922
0.013250	0.037736	0.049108	0.001452	0.094215	0.012024	0.052269	0.009903	0.096864
0.009906	0.021233	0.042173	0.000615	0.065720	0.008446	0.052646	0.008102	0.040737
0.011822	0.016952	0.070688	0.000867	0.063267	0.003715	0.030983	0.007029	0.059854
0.017908	0.038922	0.045334	0.001200	0.099908	0.016139	0.081150	0.012751	0.085185
0.013954	0.008530	0.045023	0.000529	0.042890	0.004295	0.029704	0.007582	0.041429
0.023331	0.007750	0.054726	0.000205	0.052862	0.005556	0.050358	0.008964	0.057050

2^-ΔCT	2^-ΔCT	2^-ΔCT	2^-ΔCT	2^-ΔCT	2^-ΔCT	2^-ΔCT	2^-ΔCT	2^-ΔCT
**Rel**	**Rela**	**Relb**	**Ripk1**	**Ripk2**	**Slc20a1**	**Smad3**	**Stat1**	**Tbk1**
0.019684	0.016211	0.001839	0.003528	0.024402	0.163022	0.082078	0.042193	0.082078
0.018838	0.018969	0.001772	0.005013	0.021047	0.223719	0.061629	0.046706	0.108049
0.019310	0.018017	0.002206	0.004473	0.020270	0.132632	0.094437	0.042556	0.103342
0.015120	0.014206	0.001957	0.002767	0.024563	0.200632	0.126976	0.042178	0.102424
0.019243	0.011682	0.001565	0.003196	0.024357	0.133088	0.067942	0.044516	0.085998
0.016209	0.008105	0.006142	0.005237	0.018879	0.104600	0.036726	0.023732	0.099646
0.015205	0.007926	0.004879	0.004160	0.020628	0.115882	0.026844	0.033510	0.103001
0.013585	0.008538	0.005594	0.003874	0.029526	0.093957	0.034630	0.030568	0.118105
0.015979	0.007559	0.005849	0.004341	0.025074	0.080345	0.040452	0.019402	0.096212
0.017358	0.008619	0.006310	0.005343	0.021370	0.154080	0.010465	0.034476	0.121729
0.014402	0.014807	0.001800	0.003502	0.020940	0.162939	0.067564	0.048442	0.086713
0.023153	0.016949	0.001628	0.003588	0.019335	0.185221	0.072159	0.041733	0.092611
0.021133	0.014434	0.002499	0.005068	0.025483	0.221542	0.078327	0.044987	0.084533
0.017827	0.020056	0.002050	0.005602	0.020336	0.163821	0.084799	0.049728	0.088400
0.014481	0.013605	0.002153	0.002706	0.018715	0.157161	0.064718	0.046402	0.082487
0.015501	0.010810	0.004153	0.003468	0.020739	0.125738	0.045076	0.036867	0.082956
0.004987	0.009501	0.002097	0.003502	0.016890	0.136059	0.031958	0.025248	0.052640
0.013128	0.009949	0.004422	0.003744	0.016275	0.133860	0.066930	0.036621	0.082401
0.055508	0.010812	0.001479	0.008662	0.010159	0.029134	0.009219	0.088317	0.023177
0.072344	0.014092	0.002474	0.016076	0.011526	0.035674	0.018594	0.098824	0.023373
0.022465	0.003731	0.001375	0.005734	0.004256	0.034766	0.008454	0.059286	0.014719
0.052978	0.019257	0.001679	0.005454	0.003938	0.034953	0.019124	0.105224	0.027234
0.029692	0.006329	0.000988	0.006329	0.004897	0.022659	0.007579	0.055024	0.013852
0.039749	0.014250	0.000481	0.009081	0.002626	0.022672	0.013669	0.073153	0.018933
0.019696	0.008339	0.000454	0.009062	0.003249	0.013176	0.010628	0.041351	0.013453
0.036629	0.007594	0.002035	0.005223	0.002760	0.027378	0.005598	0.063775	0.024504
0.037896	0.007803	0.000897	0.005594	0.004011	0.023653	0.011503	0.044445	0.018176
0.091626	0.014598	0.005306	0.013156	0.010911	0.024551	0.009303	0.184526	0.028006
0.084911	0.010041	0.003550	0.007878	0.008621	0.030021	0.012710	0.111267	0.024725
0.077079	0.009179	0.002082	0.005092	0.006014	0.022135	0.006766	0.088540	0.036209
0.055846	0.008654	0.000396	0.004065	0.006077	0.024648	0.011987	0.139429	0.018940
0.102007	0.017298	0.002913	0.010429	0.013385	0.023630	0.012663	0.128224	0.027332
0.038655	0.006074	0.001605	0.005179	0.002433	0.022826	0.007582	0.086378	0.022668
0.033922	0.006562	0.001053	0.005713	0.010587	0.014972	0.004211	0.077933	0.021618

2^-ΔCT	2^-ΔCT	2^-ΔCT	2^-ΔCT	2^-ΔCT	2^-ΔCT	2^-ΔCT	2^-ΔCT	2^-ΔCT
**Tlr1**	**Tlr2**	**Tlr3**	**Tlr4**	**Tlr6**	**Tlr9**	**Tnf**	**Tnfaip3**	**Tnfrsf10b**
0.000985	0.002443	0.031102	0.004623	0.004196	0.002807	0.000027	0.006630	0.004374
0.001343	0.002724	0.028355	0.004842	0.004455	0.003879	0.000030	0.005838	0.004875
0.001804	0.003098	0.023284	0.004929	0.004116	0.002516	0.000278	0.005431	0.004473
0.001600	0.003268	0.030032	0.004654	0.001657	0.003527	0.000336	0.004686	0.005769
0.001325	0.001995	0.025391	0.005228	0.003962	0.002841	0.000281	0.007445	0.003697
0.001788	0.002172	0.030883	0.004404	0.006228	0.003914	0.000188	0.004687	0.002806
0.002109	0.002168	0.048052	0.003547	0.004160	0.002783	0.000204	0.004584	0.003854
0.001370	0.001408	0.039231	0.004420	0.005913	0.003742	0.000355	0.004904	0.002401
0.002156	0.001680	0.028406	0.003779	0.008045	0.004685	0.000231	0.003832	0.003336
0.003266	0.003335	0.031072	0.003155	0.003649	0.004749	0.000358	0.004340	0.003938
0.001452	0.002864	0.030871	0.003290	0.001825	0.002986	0.000119	0.006055	0.003600
0.000955	0.001757	0.033431	0.003766	0.001286	0.003348	0.000108	0.008017	0.004150
0.000960	0.001855	0.041397	0.002364	0.001804	0.002332	0.000086	0.010567	0.006111
0.001325	0.001981	0.039015	0.002821	0.000681	0.002920	0.000102	0.010898	0.004777
0.001178	0.002094	0.025743	0.003880	0.001631	0.002687	0.000031	0.006709	0.003240
0.001938	0.001305	0.032318	0.002369	0.002817	0.002817	0.000124	0.007034	0.001872
0.001327	0.001970	0.019002	0.002654	0.001813	0.002672	0.000189	0.008989	0.003885
0.002077	0.001521	0.024668	0.002684	0.001652	0.002958	0.000211	0.009283	0.002741
0.016733	0.022233	0.002666	0.050027	0.039523	0.003930	0.001664	0.006656	0.001083
0.016758	0.024535	0.000557	0.040978	0.036423	0.008980	0.000799	0.010245	0.001502
0.027658	0.008752	0.000678	0.036749	0.024078	0.003094	0.004227	0.003433	#NULL!
0.020497	0.026124	0.000000	0.030218	0.019799	0.006094	0.001335	0.003500	0.002475
0.016936	0.006114	0.000321	0.026209	0.033405	0.008409	#NULL!	0.001593	0.000396
0.015811	0.035576	0.000000	0.078949	0.060248	0.007076	0.002163	0.006246	0.001277
0.009062	0.040221	0.000283	0.052705	0.029648	0.001959	0.001636	0.005578	0.002014
0.023669	0.045096	0.000000	0.069789	0.052890	0.000898	0.003238	0.003375	0.000930
0.009025	0.013775	0.000739	0.035604	0.020591	0.007537	0.003928	0.003874	0.000699
0.042745	0.019531	0.001915	0.024213	0.012190	0.015646	0.000423	0.010612	0.001472
0.024216	0.019132	0.000000	0.014701	0.006311	0.006625	0.000550	0.009566	#NULL!
0.025603	0.005199	0.000032	0.015544	0.004751	0.006535	0.000999	0.005890	0.001013
0.020726	0.011419	0.000000	0.012154	0.004670	0.009602	0.003539	0.003539	#NULL!
0.041142	0.014851	0.001216	0.029703	0.008589	0.015375	0.003791	0.009935	0.003210
0.017419	0.010074	0.000430	0.016594	0.006786	0.006376	0.001650	0.009270	0.000643
0.023331	0.007131	0.000142	0.019893	0.010015	0.006654	0.004124	0.004482	0.000013

2^-ΔCT	2^-ΔCT	2^-ΔCT	2^-ΔCT	2^-ΔCT	2^-ΔCT	2^-ΔCT	2^-ΔCT	2^-ΔCT
**Tnfrsf1a**	**Tnfrsf1b**	**Tnfsf10**	**Tnfsf14**	**Tollip**	**Tradd**	**Traf2**	**Traf3**	**Traf5**
0.018239	0.006863	0.004404	0.000238	0.167606	0.019684	0.015878	0.047469	0.004254
0.023032	0.006433	0.005920	0.000459	0.167213	0.029765	0.016744	0.047031	0.003496
0.023123	0.010065	0.002348	0.000240	0.170223	0.030938	0.013654	0.053124	0.004631
0.018232	0.006183	0.003996	0.000403	0.188498	0.022760	0.014010	0.047452	0.004818
0.015098	0.005681	0.005488	0.000850	0.161595	0.024357	0.011363	0.050083	0.002340
0.012630	0.004373	0.007506	0.007774	0.109042	0.027450	0.006629	0.017493	0.002264
0.013609	0.004131	0.005924	0.006305	0.109631	0.027598	0.005565	0.022417	0.002109
0.012414	0.004607	0.006840	0.006653	0.103532	0.025704	0.006935	0.020307	0.002977
0.014302	0.004136	0.005611	0.006580	0.116820	0.020367	0.006400	0.021828	0.002425
0.016651	0.004400	0.008923	0.010109	0.120889	0.039329	0.008500	0.024043	0.002325
0.016203	0.006140	0.003626	0.000606	0.148898	0.024730	0.013070	0.058008	0.002111
0.011182	0.004417	0.003714	0.000060	0.193087	0.018676	0.011985	0.038137	0.002815
0.020413	0.004321	0.003710	0.000043	0.187590	0.019046	0.014137	0.046897	0.003684
0.021347	0.005922	0.003961	0.000057	0.174366	0.024521	0.012261	0.051126	0.002961
0.016520	0.005337	0.002961	0.000373	0.159355	0.023688	0.013325	0.056341	0.003023
0.010514	0.003929	0.005043	0.004577	0.124869	0.018054	0.008659	0.033925	0.001938
0.012450	0.003027	0.003267	0.001124	0.145824	0.019267	0.009973	0.043655	0.002310
0.011039	0.004242	0.004126	0.002353	0.134791	0.016388	0.008661	0.032776	0.002419
0.105758	0.060322	0.002629	0.069774	0.079046	0.022543	0.011750	0.002353	0.019221
0.106654	0.065200	0.003647	0.088450	0.085437	0.024706	0.009827	0.006712	0.056760
0.065327	0.024078	0.002158	0.066239	0.059698	0.016791	0.010627	0.006363	0.011389
0.143741	0.108183	0.004715	0.049430	0.116754	0.022901	0.011293	0.007554	0.027423
0.068689	0.073619	0.001015	0.088770	0.088157	0.013948	0.012484	0.008951	0.016022
0.190395	0.113997	0.004799	0.140347	0.075733	0.028897	0.011817	0.007689	0.016255
0.117773	0.000000	0.003226	0.101116	0.095001	0.013928	0.008111	0.003890	0.012639
0.158125	0.100770	0.005521	0.130230	0.087725	0.022548	0.009285	0.002092	0.008907
0.080113	0.062855	0.002521	0.086460	0.068307	0.016381	0.011745	0.004770	0.011745
0.065694	0.087893	0.005380	0.032395	0.053731	0.025066	0.013156	0.007609	0.040721
0.045503	0.071402	0.001383	0.039339	0.127813	0.017727	0.022751	0.007199	0.031953
0.030448	0.054127	0.004372	0.043661	0.064815	0.012802	0.009975	0.006445	0.026141
0.044121	0.070688	0.003793	0.047948	0.120541	0.024648	0.007639	0.004180	0.008075
0.042006	0.054287	0.005667	0.032503	0.053539	0.024463	0.023630	0.013019	0.055044
0.025503	0.051718	0.004933	0.034838	0.053172	0.018930	0.004540	0.004063	0.010575
0.036610	0.087073	0.003590	0.037379	0.059473	0.019349	0.010513	0.006383	0.012765

2^-ΔCT	2^-ΔCT	2^-ΔCT	2^-ΔCT	2^-ΔCT	2^-ΔCT	2^-ΔCT	2^-ΔCT	2^-ΔCT
**Traf6**	**Zap70**	**Alb**	**Als2**	**Aox1**	**Apc**	**Apoe**	**Atr**	**Cat**
0.033104	0.006863	0.001014	0.106061	0.009052	0.302754	3.417521	0.049555	0.127813
0.037675	0.005298	0.000903	0.079717	0.005705	0.332079	3.150710	0.046244	0.123180
0.039706	0.007788	0.000795	0.074413	0.005949	0.284211	2.676149	0.042898	0.116653
0.038013	0.008331	0.001312	0.108437	0.006911	0.240360	3.047744	0.049596	0.134815
0.035169	0.005412	0.000765	0.089238	0.004930	0.244211	2.199354	0.035482	0.115642
0.036221	0.004753	0.005098	0.078490	0.007181	0.432827	3.736869	0.078355	0.162679
0.035176	0.003672	0.004512	0.081274	0.007736	0.395830	3.259427	0.069161	0.173368
0.034870	0.004359	0.005525	0.109625	0.008695	0.540762	4.698896	0.096435	0.229140
0.043961	0.004341	0.000392	0.070140	0.009036	0.392823	2.666166	0.067129	0.135266
0.028394	0.004950	0.005353	0.097114	0.009371	0.449209	3.715495	0.088251	0.206377
0.038537	0.010398	0.000670	0.063927	0.005891	0.215226	2.564419	0.031845	0.095463
0.034133	0.006467	0.000207	0.082217	0.007551	0.274554	2.977613	0.034683	0.119064
0.030941	0.008233	0.000238	0.111531	0.007602	0.337657	3.216231	0.023855	0.125887
0.042694	0.012781	0.000177	0.096481	0.008080	0.290549	3.324336	0.038134	0.124573
0.039290	0.010099	0.001058	0.080571	0.005543	0.276076	3.056466	0.035464	0.117901
0.028527	0.008540	0.001609	0.092409	0.006899	0.317491	3.038310	0.046451	0.145706
0.053746	0.008214	0.000953	0.012246	0.001107	0.047671	0.260853	0.006478	0.011469
0.031660	0.006210	0.001477	0.076039	0.006309	0.300698	2.921429	0.044446	0.107023
#NULL!	0.017810	1.512385	0.045960	0.000000	0.003703	0.117032	0.007965	0.968906
0.006901	0.017349	0.001483	0.026440	0.000000	0.003204	0.085063	0.008183	1.754050
#NULL!	0.008454	0.000091	0.059396	0.000000	0.007002	0.034574	0.005839	1.012499
0.005342	0.024039	0.000272	0.028054	0.000000	0.003552	0.122866	0.002712	2.961159
0.009527	0.011648	0.000046	0.018579	0.000000	0.005543	0.074090	0.002590	1.065672
0.005950	0.020152	0.000158	0.019409	0.000000	0.003378	0.103887	0.005038	1.111356
0.002602	0.009848	0.000218	0.017837	0.000267	0.002707	0.079855	0.003770	1.424482
#NULL!	0.010592	0.000517	0.043269	0.000000	0.006841	0.076572	0.002107	1.544708
0.003901	0.007642	0.000146	0.031818	0.000000	0.003096	0.086863	0.003376	1.107864
0.006223	0.008099	0.000020	0.016258	0.000000	0.004590	0.160756	0.003453	3.229288
0.007824	0.011777	0.000345	0.015947	0.000000	0.006322	0.088871	0.001825	7.076050
#NULL!	0.005972	0.000520	0.034655	0.000000	0.003656	0.061925	0.007428	4.367108
0.002485	0.009022	0.000689	0.019491	0.000000	0.004736	0.060864	0.003866	5.902578
0.010285	0.008014	0.000172	0.020784	0.000000	0.001489	0.114114	0.010976	4.101874
0.000764	0.015164	0.002627	0.030314	0.000000	0.004724	0.120720	0.002828	2.963001
0.005184	0.007332	0.000445	0.026281	0.000000	0.003857	0.117062	0.002360	1.988118

2^-ΔCT	2^-ΔCT	2^-ΔCT	2^-ΔCT	2^-ΔCT	2^-ΔCT	2^-ΔCT	2^-ΔCT	2^-ΔCT
**Ccs**	**Ctsb**	**Cyba**	**Cygb**	**Dnm2**	**Duox1**	**Ehd2**	**Epx**	**Ercc2**
0.040397	0.602074	0.028457	0.082625	0.042009	0.004118	0.014634	0.002579	0.073861
0.043890	0.573880	0.023076	0.122057	0.034113	0.003133	0.009761	0.002043	0.057090
0.042545	0.503732	0.022009	0.099391	0.031350	0.002076	0.007490	0.000925	0.046023
0.034498	0.583694	0.028133	0.082309	0.043725	0.003369	0.008871	0.001528	0.076529
0.026468	0.508199	0.022063	0.098877	0.031704	0.002856	0.007124	0.001085	0.049669
0.033546	0.505302	0.076586	0.075290	0.055903	0.002601	0.012952	0.002076	0.049524
0.022313	0.414715	0.084225	0.089790	0.047624	0.002839	0.011042	0.003156	0.051120
0.036219	0.563885	0.083899	0.086895	0.067223	0.002840	0.015811	0.002850	0.072522
0.030182	0.340890	0.098063	0.051498	0.055745	0.003105	0.011453	0.002574	0.042355
0.022871	0.477833	0.095930	0.115290	0.068247	0.004633	0.016667	0.004648	0.072629
0.025762	0.340438	0.017309	0.069885	0.024993	0.001862	0.004827	0.002045	0.035252
0.026991	0.559356	0.018393	0.060028	0.039238	0.002432	0.008453	0.000517	0.044338
0.036793	0.667321	0.021009	0.097243	0.049785	0.003306	0.009254	0.000663	0.041025
0.038423	0.546081	0.022239	0.082475	0.039675	0.002676	0.012845	0.000290	0.039380
0.029515	0.531059	0.021429	0.124999	0.031242	0.002469	0.008318	0.001473	0.045624
0.020313	0.410229	0.046138	0.058446	0.059334	0.003223	0.009835	0.001550	0.049398
0.001701	0.037814	0.011459	0.006037	0.014116	0.001292	0.003328	0.000843	0.032015
0.019876	0.405348	0.045275	0.054983	0.052961	0.002624	0.009520	0.001332	0.050334
0.021363	0.171228	0.298968	0.000515	0.048328	0.000000	0.000952	0.000000	0.007839
0.017974	0.288099	0.291290	0.000335	0.053125	0.000000	0.000065	0.000000	0.007839
0.028137	0.212096	0.303170	0.000379	0.038474	0.000000	0.000000	0.000000	0.010145
0.024420	0.631102	0.327108	0.000489	0.047242	0.000000	0.000000	0.000000	0.005181
0.027969	0.200624	0.271592	0.000226	0.037233	0.000000	0.000000	0.000000	0.008826
0.024349	0.220507	0.380760	0.000195	0.050420	0.000000	0.000000	0.000010	0.006113
0.021266	0.225307	0.282196	0.001533	0.039377	0.000000	0.000000	0.001064	0.007290
0.026956	0.218581	0.291074	0.001897	0.043045	0.000000	0.000000	0.000279	0.008603
0.024822	0.193378	0.302577	0.001176	0.050990	0.000000	0.000050	0.000858	0.005542
0.039463	0.354099	0.217466	0.000254	0.042884	0.000000	0.000961	0.000000	0.009662
0.035266	0.568855	0.246475	0.000629	0.037802	0.000000	0.000483	0.000000	0.006580
0.030808	0.334856	0.170472	0.002698	0.022864	0.000659	0.000650	0.000244	0.009760
0.038384	0.488934	0.182756	0.001938	0.027353	0.000000	0.000689	0.000992	0.002099
0.029276	0.375053	0.331499	0.001124	0.042151	0.000000	0.002076	0.000000	0.009100
0.030996	0.309804	0.197199	0.002146	0.033529	0.000000	0.000000	0.001024	0.005634
0.021266	0.221911	0.209011	0.001783	0.025088	0.000000	0.001091	0.000021	0.006940

2^-ΔCT	2^-ΔCT	2^-ΔCT	2^-ΔCT	2^-ΔCT	2^-ΔCT	2^-ΔCT	2^-ΔCT	2^-ΔCT
**Ercc6**	**Fancc**	**Fmo2**	**Fth1**	**Gclc**	**Gclm**	**Gpx1**	**Gpx2**	**Gpx3**
0.055991	0.010795	0.004806	10.941519	0.214460	0.235315	0.388274	0.001267	0.020078
0.039146	0.011436	0.004762	10.170256	0.200220	0.267346	0.518467	0.001062	0.036248
0.040501	0.009308	0.002830	9.563217	0.172150	0.226240	0.382339	0.001146	0.038446
0.061389	0.009825	0.003380	9.930669	0.193674	0.230471	0.338398	0.001794	0.013186
0.037562	0.008522	0.003111	9.148233	0.185347	0.212305	0.423419	0.001155	0.031660
0.067267	0.010400	0.004581	18.981101	0.233024	0.245531	0.710309	0.002447	0.031883
0.062070	0.009414	0.004479	16.459640	0.245909	0.262519	0.784869	0.002343	0.093739
0.084203	0.010747	0.004862	19.972587	0.319594	0.321698	0.994076	0.002560	0.028109
0.069220	0.007252	0.003507	15.415850	0.210674	0.181220	0.493523	0.002660	0.019343
0.059046	0.016341	0.004626	21.322806	0.332306	0.332421	1.251289	0.004417	0.075696
0.028073	0.007272	0.001671	8.761655	0.133432	0.168196	0.330275	0.001013	0.020899
0.032438	0.011923	0.002287	12.979557	0.195104	0.206404	0.307132	0.000310	0.022856
0.038970	0.013528	0.002158	11.783017	0.196849	0.216724	0.383630	0.000810	0.022854
0.034021	0.009137	0.004233	13.934547	0.188921	0.202926	0.333199	0.000392	0.017391
0.038051	0.009240	0.001294	9.912272	0.170510	0.227564	0.490869	0.000983	0.021372
0.047375	0.009648	0.002511	13.636568	0.191591	0.199794	0.405755	0.002069	0.008016
0.020977	0.001686	0.000862	1.462932	0.018925	0.024613	0.042197	0.001154	0.008050
0.050190	0.008719	0.002528	11.313509	0.188880	0.175457	0.362464	0.001767	0.010076
0.000630	0.001504	0.002872	23.126082	0.077416	1.601747	99.995009	0.000033	0.001525
0.001853	0.002959	0.000000	25.143057	0.074488	1.881469	69.431546	0.000000	0.004435
0.001809	0.006658	0.000000	38.459133	0.076534	2.161672	########	0.000026	0.003647
0.004576	0.002636	0.000856	23.855340	0.060794	2.699945	63.072400	0.000024	0.005075
0.002673	0.003790	0.000000	18.846174	0.067386	1.377639	61.347142	0.000033	0.003161
0.001069	0.002356	0.000553	17.673417	0.039949	1.245541	64.814637	0.000025	0.004821
0.002144	0.001491	0.000000	17.416949	0.043993	1.503444	63.846387	0.000160	0.005301
0.000607	0.001180	0.000000	23.031176	0.069934	1.806029	########	0.000304	0.001042
0.003011	0.004683	0.000000	19.972450	0.059514	1.580353	86.522993	0.000053	0.000818
0.003151	0.003439	0.000000	14.956286	0.046785	1.795154	########	0.000000	0.004513
0.002342	0.003471	0.000000	22.302849	0.064506	3.913056	93.271666	0.000039	0.003883
0.002059	0.002454	0.000000	23.274216	0.058920	2.536129	########	0.001100	0.001855
0.001026	0.002995	0.000000	20.961811	0.044408	2.989851	########	0.000148	0.002999
0.004774	0.004346	0.000000	17.212521	0.045513	2.286157	########	0.000066	0.002878
0.001930	0.001420	0.000000	19.815716	0.049180	2.307401	########	0.000182	0.007666
0.000121	0.002774	0.000000	19.813509	0.042579	1.710540	########	0.000084	0.002430

2^-ΔCT	2^-ΔCT	2^-ΔCT	2^-ΔCT	2^-ΔCT	2^-ΔCT	2^-ΔCT	2^-ΔCT	2^-ΔCT
**Gpx4**	**Gpx5**	**Gpx6**	**Gpx7**	**Gsr**	**Gss**	**Gstk1**	**Gstp1**	**Hmox1_bi**
0.872103	0.004907	0.000666	0.006760	0.079282	0.091660	0.041022	0.795880	0.013672
0.851438	0.004642	0.000275	0.006886	0.078070	0.087377	0.041515	0.632559	0.007681
0.717291	0.002136	0.000141	0.004920	0.074764	0.067622	0.041915	0.569925	0.008636
0.765474	0.005954	0.000559	0.006614	0.070722	0.088582	0.037613	0.702349	0.017461
0.758319	0.003813	0.000523	0.004648	0.063335	0.064762	0.033349	0.578708	0.006902
1.095766	0.008055	0.001577	0.007398	0.063005	0.096382	0.052283	0.628419	0.018630
1.167685	0.007940	0.001118	0.008906	0.061963	0.086128	0.063303	0.700737	0.018880
1.200433	0.008528	0.001942	0.009829	0.082745	0.114197	0.059233	0.764194	0.021801
0.990841	0.007864	0.001250	0.008177	0.057761	0.063823	0.037013	0.544022	0.020409
1.456490	0.010293	0.001835	0.009716	0.086858	0.149579	0.085194	0.908558	0.018453
0.662852	0.004627	0.000462	0.004798	0.049661	0.056876	0.036183	0.505294	0.006994
0.923607	0.000093	0.000092	0.004904	0.068853	0.061727	0.041459	0.745920	0.004404
0.950640	0.000105	0.000429	0.006492	0.085585	0.071785	0.046960	0.663478	0.003227
0.890082	0.000080	0.000062	0.005744	0.064302	0.065950	0.048739	0.678569	0.005242
0.764522	0.004153	0.000420	0.005222	0.062848	0.058632	0.037414	0.587245	0.006049
0.869017	0.004560	0.000749	0.008052	0.057838	0.057939	0.043607	0.698045	0.047642
0.484464	0.001408	0.000257	0.004209	0.034248	0.049473	0.013981	0.162597	0.034643
0.934690	0.003047	0.000778	0.007273	0.071009	0.061242	0.038003	0.682841	0.048512
2.974686	0.000075	0.000000	0.001052	0.320237	0.020855	0.014345	0.091295	0.002384
2.290462	0.000012	0.000000	0.000000	0.386120	0.016895	0.012935	0.092452	0.005544
5.124251	0.000069	0.000000	0.000000	0.348857	0.015998	0.016498	0.058417	0.002764
2.000968	0.000028	0.000000	0.000000	0.464732	0.014975	0.019002	0.092058	0.006516
2.886565	0.000000	0.000000	0.000210	0.229132	0.014513	0.013179	0.068709	0.002964
2.094587	0.000017	0.000000	0.000179	0.583096	0.023407	0.012885	0.074274	0.006411
2.226819	0.000151	0.000000	0.000013	0.386418	0.011388	0.015825	0.064295	0.005680
3.150428	0.000236	0.000000	0.000694	0.552339	0.018593	0.009225	0.068458	0.008015
2.911753	0.000167	0.000000	0.000631	0.240991	0.020593	0.012574	0.077087	0.002335
1.438380	0.000000	0.000000	0.000000	0.174870	0.018752	0.007412	0.092709	0.005001
1.684044	0.000075	0.000000	0.000099	0.213864	0.019885	0.011327	0.107894	0.003514
2.167250	0.000549	0.000128	0.000141	0.144453	0.013011	0.020580	0.080266	0.006528
1.879675	0.000091	0.000000	0.000025	0.157826	0.014652	0.007337	0.102888	0.002884
1.615982	0.000163	0.000000	0.000533	0.179878	0.016974	0.015385	0.107682	0.003465
2.494342	0.000185	0.000000	0.000020	0.186925	0.010193	0.015632	0.108720	0.006317
2.440763	0.000172	0.000000	0.000032	0.169542	0.016923	0.014437	0.083800	0.004684

2^-ΔCT	2^-ΔCT	2^-ΔCT	2^-ΔCT	2^-ΔCT	2^-ΔCT	2^-ΔCT	2^-ΔCT	2^-ΔCT
**Hspa1a**	**Idh1**	**Ift172**	**Il19**	**Il22**	**Krt1**	**Lpo**	**Mb**	**Mpo**
0.028221	0.186031	0.091549	0.000310	0.000354	0.038510	0.002995	0.005071	0.000420
0.015290	0.132581	0.073747	0.000235	0.001942	0.035371	0.004777	0.009969	0.000280
0.031385	0.125503	0.074799	0.000065	0.001368	0.021091	0.002680	0.002944	0.000364
0.027172	0.122426	0.091198	0.000348	0.000214	0.028142	0.002938	0.002936	0.000865
0.027301	0.114373	0.060062	0.000194	0.000176	0.017046	0.003030	0.003439	0.000362
0.031481	0.190187	0.055446	0.001170	0.001223	0.024198	0.004212	0.010363	0.002093
0.040389	0.194914	0.057566	0.001449	0.001247	0.022344	0.006210	0.007405	0.002341
0.054587	0.175170	0.058023	0.001453	0.001619	0.030612	0.003855	0.011304	0.002272
0.039615	0.189049	0.047750	0.001789	0.001833	0.027272	0.002646	0.007255	0.001520
0.032626	0.200807	0.066846	0.001304	0.001637	0.023973	0.005896	0.017512	0.002210
0.014494	0.098060	0.055472	0.000193	0.000257	0.034197	0.003253	0.003814	0.000250
0.024750	0.115400	0.081384	0.000123	0.003531	0.002067	0.000836	0.002080	0.000210
0.028680	0.125120	0.085000	0.000046	0.000340	0.004475	0.001666	0.002270	0.000133
0.070941	0.122507	0.070800	0.000073	0.000047	0.001767	0.001130	0.003099	0.000268
0.025626	0.096233	0.062462	0.000150	0.000272	0.034793	0.004214	0.003004	0.000468
0.025438	0.110991	0.064315	0.000639	0.003502	0.058633	0.001483	0.003201	0.001177
0.003003	0.045070	0.050064	0.000210	0.000112	0.020125	0.001883	0.002099	0.000484
0.023646	0.119340	0.074205	0.000386	0.000445	0.051449	0.001269	0.002390	0.000862
0.000000	0.035056	0.001365	0.000000	0.000000	0.000047	0.000000	0.025238	0.000756
0.000832	0.023499	0.002729	0.000591	0.000000	0.000037	0.000000	0.000000	0.000175
0.000341	0.025893	0.001665	0.002747	0.000000	0.000119	0.000000	0.000000	0.003008
0.000000	0.023505	0.001990	0.000000	0.000000	0.000049	0.000000	0.000000	0.000556
0.000000	0.021018	0.002210	0.000955	0.000000	0.000029	0.000000	0.000000	0.000379
0.000870	0.021697	0.001798	0.001754	0.000000	0.000026	0.000000	0.112322	0.000000
0.000346	0.031925	0.002690	0.000643	0.000019	0.009141	0.000000	0.049592	0.000786
0.001712	0.010841	0.004933	0.000000	0.000000	0.012592	0.000000	0.019829	0.000505
0.000000	0.015515	0.002142	0.000614	0.000000	0.003457	0.000105	0.000000	0.000000
0.000000	0.035608	0.004930	0.000573	0.000000	0.000017	0.000000	0.000000	0.000879
0.001035	0.015664	0.002766	#NULL!	0.000000	0.000123	0.000000	0.003105	0.000000
0.003795	0.024986	0.009447	#NULL!	0.000026	0.019887	0.000021	0.000000	0.000039
0.000289	0.029719	0.004324	0.000000	0.000000	0.003095	0.000000	0.000000	0.000000
0.000000	0.045024	0.007455	0.000000	0.000000	0.001076	0.000000	0.000000	0.000878
0.000984	0.040971	0.002908	0.000000	0.000027	0.009170	0.000078	0.000000	0.002364
0.000000	0.045232	0.006834	0.000000	0.000000	0.013345	0.000000	0.000000	0.000510

2^-ΔCT	2^-ΔCT	2^-ΔCT	2^-ΔCT	2^-ΔCT	2^-ΔCT	2^-ΔCT	2^-ΔCT	2^-ΔCT
**Ncf1**	**Ncf2**	**Ngb**	**Nos2**	**Nox1**	**Nox4**	**Noxa1**	**Noxo1**	**Nqo1**
0.011332	0.025805	0.006791	0.262872	0.001699	0.000735	0.014942	0.006362	0.018147
0.006519	0.017259	0.009973	0.187720	0.001829	0.000907	0.014824	0.005357	0.015626
0.008074	0.017993	0.009644	0.237146	0.000730	0.000951	0.008922	0.002863	0.015429
0.011081	0.021845	0.005433	0.190903	0.001278	0.000139	0.014286	0.006016	0.016902
0.006959	0.013958	0.011406	0.119357	0.000845	0.000954	0.011590	0.003884	0.011635
0.008770	0.024110	0.003929	0.165295	0.002071	0.001073	0.035992	0.012869	0.017477
0.011093	0.023410	0.009703	0.155946	0.001402	0.001520	0.039213	0.012683	0.018809
0.013744	0.022345	0.006130	0.201035	0.001980	0.000370	0.043484	0.014483	0.016800
0.007572	0.019850	0.002103	0.176541	0.003327	0.000471	0.037688	0.013087	0.018018
0.008942	0.026802	0.012874	0.180413	0.002154	0.001847	0.044388	0.013737	0.018705
0.006559	0.015005	0.007831	0.255990	0.000981	0.000332	0.012854	0.003237	0.014052
0.008689	0.013751	0.003586	0.257976	0.000719	0.000215	0.000281	0.003482	0.013565
0.012230	0.018670	0.007920	0.262019	0.000828	0.000403	0.000537	0.003953	0.019083
0.010528	0.017395	0.005142	0.298594	0.000335	0.000638	0.000339	0.003667	0.015918
0.006462	0.016841	0.007914	0.315824	0.000814	0.000578	0.012108	0.004234	0.011034
0.009569	0.019396	0.002277	0.296590	0.001588	0.000238	0.018636	0.007634	0.013323
0.007680	0.013096	0.004555	0.243955	0.000431	0.000383	0.004208	0.005042	0.016347
0.008389	0.019932	0.003319	0.418056	0.000789	0.000202	0.013617	0.006630	0.013190
0.069027	0.229394	0.000000	0.002198	0.000000	0.000000	0.000062	0.000053	0.026751
0.072225	0.226555	0.000000	0.005515	0.000846	0.000000	0.000017	0.002322	0.017028
0.042747	0.173446	0.000058	0.010437	0.000000	0.000000	0.000000	0.000419	0.032745
0.117415	0.305199	0.000000	0.006394	0.000000	0.000000	0.000000	0.001547	0.048882
0.066227	0.192834	0.000000	0.001337	0.000000	0.000000	0.000000	0.000014	0.017435
0.185363	0.426120	0.000000	0.002907	0.000000	0.000000	0.000037	0.000015	0.019679
0.088513	0.310619	0.000000	0.138482	0.000000	0.000000	0.000165	0.000578	0.021966
0.060055	0.260607	0.000000	0.146539	0.000000	0.000000	0.000252	0.000193	0.026230
0.065336	0.229528	0.000000	0.029926	0.000000	0.000000	0.000127	0.000643	0.016276
0.092019	0.142563	0.000000	0.001316	0.000966	0.000000	#NULL!	0.001149	0.024459
0.070643	0.139022	0.000000	0.009570	0.000000	0.000000	0.000027	0.001605	0.020938
0.045039	0.084248	0.000078	0.155299	0.000000	0.000000	0.000180	0.001385	0.038384
0.047669	0.084729	0.000019	0.118239	0.000000	0.000000	0.000083	0.000608	0.037970
0.127196	0.143122	0.000000	0.015581	0.000000	0.000000	0.000056	0.001468	0.023434
0.073225	0.094780	0.000000	0.132787	0.000000	0.000000	0.000322	0.000631	0.030163
0.063437	0.080993	0.000057	0.136012	0.000000	0.000000	0.000085	0.000976	0.012671

2^-ΔCT	2^-ΔCT	2^-ΔCT	2^-ΔCT	2^-ΔCT	2^-ΔCT	2^-ΔCT	2^-ΔCT	2^-ΔCT
**Park7**	**Prdx1**	**Prdx2**	**Prdx3**	**Prdx4**	**Prdx5**	**Prdx6**	**Prnp**	**Psmb5**
0.351208	0.029182	0.677528	0.204946	0.063264	0.588533	0.189887	0.515226	0.768810
0.309331	0.036056	0.567508	0.156640	0.083760	0.553969	0.158638	0.552344	0.656119
0.282937	0.031950	0.500099	0.151542	0.050219	0.412750	0.171241	0.487915	0.673505
0.344237	0.033851	0.623969	0.193739	0.049517	0.451700	0.164845	0.353824	0.704238
0.303061	0.026026	0.470435	0.133105	0.050538	0.389903	0.109610	0.258612	0.535619
0.518674	0.037456	0.658083	0.216281	0.079340	0.532360	0.158909	0.329453	0.846039
0.571470	0.041498	0.726640	0.207284	0.094349	0.566749	0.152427	0.243964	0.909291
0.572336	0.037791	0.895202	0.228022	0.089381	0.694356	0.173511	0.268930	0.878314
0.488748	0.032873	0.560080	0.152724	0.056635	0.464688	0.154767	0.293237	0.930370
0.668730	0.052320	1.039178	0.279032	0.156872	0.783723	0.143946	0.211341	0.873243
0.219989	0.029875	0.436845	0.128747	0.051818	0.359811	0.140674	0.302754	0.480094
0.268405	0.050037	0.539814	0.202774	0.084339	0.486916	0.226806	0.515442	0.502925
0.273552	0.052207	0.544579	0.212087	0.083896	0.546389	0.219405	0.563742	0.496136
0.268505	0.042178	0.544554	0.190850	0.116671	0.521408	0.245136	0.775665	0.535630
0.254954	0.026425	0.476876	0.126857	0.047806	0.381384	0.147060	0.293436	0.540658
0.374931	0.028620	0.585333	0.179873	0.054334	0.434953	0.191834	0.214415	0.635993
0.504856	0.043412	0.866018	0.249177	0.082272	0.731131	0.272563	0.222025	0.621511
0.399870	0.034533	0.629152	0.204234	0.057456	0.499685	0.213594	0.356800	0.721764
0.024736	0.006083	3.189881	0.116554	0.099382	0.268025	0.307629	0.006859	0.190048
0.029689	0.003113	2.785926	0.111790	0.055848	0.234062	0.355474	0.011136	0.177062
0.024085	0.003099	2.621887	0.150921	0.057723	0.257667	0.415649	0.006753	0.218939
0.023988	0.010628	3.123748	0.113722	0.031012	0.262469	0.357610	0.017577	0.156025
0.027771	0.003258	1.741898	0.108053	0.056980	0.202441	0.298355	0.003556	0.186428
0.025804	0.003457	2.370300	0.070027	0.053131	0.409330	0.205638	0.002400	0.141688
0.019022	0.003802	2.215563	0.081446	0.042985	0.325292	0.274061	0.007400	0.156710
0.019368	0.003770	4.000946	0.104752	0.063314	0.337146	0.250369	0.005066	0.155164
0.021273	0.003045	2.604615	0.098782	0.065825	0.230523	0.268143	0.007366	0.169442
0.029071	0.003808	4.109856	0.085294	0.057806	0.235930	0.234264	0.010627	0.115077
0.020098	0.004719	5.523086	0.098913	0.034576	0.213717	0.359684	0.016520	0.105540
0.020709	0.004410	5.564218	0.116118	0.055408	0.215360	0.264368	0.010031	0.108474
0.020212	0.003649	4.368463	0.126519	0.034698	0.161474	0.300909	0.028153	0.091700
0.028087	0.004081	3.715171	0.106048	0.045422	0.243095	0.251248	0.008118	0.117550
0.024241	0.005687	5.856914	0.095877	0.043385	0.220236	0.200872	0.009124	0.098028
0.029433	0.005107	4.514231	0.085901	0.040026	0.332452	0.214333	0.002889	0.122247

2^-ΔCT	2^-ΔCT	2^-ΔCT	2^-ΔCT	2^-ΔCT	2^-ΔCT	2^-ΔCT	2^-ΔCT	2^-ΔCT
**Ptgs1**	**Ptgs2**	**Rag2**	**Recql4**	**Scd1**	**Serpinb1b**	**Slc38a1**	**Sod1**	**Sod2**
0.030845	0.049755	0.000701	0.003574	0.313125	0.012711	0.193899	0.403123	0.649275
0.024683	0.015862	0.000518	0.003198	0.410245	0.022458	0.257705	0.378221	0.533827
0.022477	0.045397	0.000270	0.002964	0.255231	0.009625	0.158451	0.290087	0.405166
0.026472	0.055880	0.001046	0.004740	0.289147	0.005828	0.191623	0.354992	0.587167
0.017897	0.036357	0.000629	0.002770	0.235792	0.013773	0.138675	0.319724	0.403538
0.041258	0.043588	0.002619	0.010271	0.418460	0.023372	0.243155	0.449496	0.486452
0.039411	0.043699	0.003859	0.010459	0.486564	0.021930	0.268112	0.497922	0.530416
0.044070	0.048137	0.004166	0.009722	0.354267	0.016667	0.261811	0.559291	0.579265
0.039818	0.058304	0.003533	0.009904	0.298205	0.009195	0.153520	0.441518	0.418099
0.046760	0.019714	0.003441	0.005478	0.628607	0.049724	0.388266	0.706644	0.671437
0.016101	0.028312	0.000687	0.003064	0.253817	0.018544	0.139692	0.318935	0.394903
0.017375	0.036541	0.000058	0.002582	0.265130	0.014064	0.167830	0.387471	0.505433
0.021848	0.034314	0.000070	0.003351	0.269979	0.024297	0.203105	0.539429	0.580835
0.017627	0.050454	0.000110	0.003002	0.275513	0.017240	0.169304	0.384012	0.492005
0.018364	0.030798	0.000656	0.003254	0.246862	0.011081	0.142727	0.266620	0.370153
0.028076	0.052931	0.001470	0.005839	0.254625	0.010178	0.181702	0.411750	0.433782
0.020917	0.046433	0.000232	0.003768	0.395883	0.015249	0.316472	0.833219	0.818744
0.024768	0.046341	0.001093	0.004479	0.190825	0.005820	0.144450	0.405257	0.462217
0.047140	0.002597	0.000000	0.000222	0.086507	0.023527	0.023659	0.166871	0.201890
0.056109	0.003293	0.000000	0.000075	0.039842	0.025366	0.034043	0.197353	0.174118
0.045605	0.000000	0.000000	0.000237	0.029574	0.016527	0.009834	0.149088	0.125997
0.141285	0.000000	0.000000	0.001950	0.020426	0.009554	0.032476	0.098606	0.241716
0.037356	0.000965	0.000000	0.001429	0.039185	0.020385	0.012516	0.111496	0.107611
0.033015	0.000014	0.000000	0.000409	0.029435	0.016723	0.010484	0.124104	0.148585
0.084907	0.000049	0.000000	0.001882	0.011467	0.025153	0.008971	0.117671	0.128233
0.039059	0.000589	0.000000	0.002848	0.025266	0.020873	0.006508	0.135549	0.195318
0.041677	0.002755	0.000000	0.000996	0.039841	0.025297	0.010799	0.146307	0.126702
0.048544	0.000583	#NULL!	0.000451	0.100558	0.027937	0.039743	0.148689	0.198583
0.117929	0.000410	#NULL!	0.000601	0.068675	0.033033	0.050201	0.086874	0.226754
0.061800	0.000264	0.000088	0.005120	0.086347	0.044125	0.025581	0.118217	0.244636
0.102923	0.000905	0.000000	0.000809	0.051255	0.019497	0.018219	0.120782	0.254221
0.061586	0.001191	0.000000	0.000989	0.144585	0.056539	0.051012	0.131838	0.215401
0.040875	0.000055	0.000000	0.004133	0.076230	0.023066	0.009797	0.145543	0.202846
0.033307	0.000018	0.000000	0.001906	0.066653	0.026383	0.011302	0.174300	0.160025

2^-ΔCT	2^-ΔCT	2^-ΔCT	2^-ΔCT	2^-ΔCT	2^-ΔCT	2^-ΔCT	2^-ΔCT	2^-ΔCT
**Sod3**	**Sqstm1**	**Srxn1**	**Tpo**	**Txn1**	**Txnip**	**Txnrd1**	**Txnrd2**	**Txnrd3**
0.022143	0.473308	0.119507	0.001421	0.003937	0.063068	0.102881	0.036759	0.008017
0.021609	0.507558	0.140542	0.002203	0.002820	0.051704	0.092957	0.031573	0.009274
0.011188	0.426754	0.098407	0.001004	0.001798	0.049158	0.078743	0.030172	0.004600
0.021210	0.445878	0.118875	0.001694	0.004344	0.030341	0.099785	0.037985	0.006132
0.013418	0.260217	0.073603	0.001438	0.002388	0.027475	0.061093	0.022341	0.004622
0.017452	0.303336	0.104049	0.003071	0.014661	0.030937	0.096490	0.033990	0.006372
0.017407	0.325687	0.089016	0.004081	0.013465	0.041009	0.088260	0.038905	0.006096
0.017192	0.331423	0.092123	0.003572	0.016196	0.038184	0.094077	0.045018	0.004193
0.016726	0.286446	0.072110	0.003438	0.015700	0.051766	0.073185	0.037214	0.003800
0.026824	0.362553	0.113685	0.003768	0.018999	0.038662	0.136270	0.046250	0.007137
0.009768	0.307961	0.069941	0.001160	0.002257	0.035558	0.056780	0.024445	0.004139
0.016794	0.469207	0.077153	0.000286	0.001500	0.054345	0.063544	0.020869	0.005721
0.020756	0.454766	0.083580	0.000909	0.001960	0.052627	0.085046	0.023928	0.008096
0.023537	0.438330	0.068719	0.000457	0.001683	0.085569	0.075820	0.023791	0.006528
0.014642	0.379849	0.077905	0.001592	0.002413	0.023272	0.057996	0.026030	0.004949
0.018809	0.417738	0.074703	0.001343	0.006501	0.043739	0.064123	0.027592	0.003277
0.046059	0.413827	0.087668	0.000932	0.001985	0.066344	0.121313	0.037481	0.007772
0.026338	0.467026	0.076068	0.001288	0.004841	0.039025	0.076889	0.026682	0.004370
0.001287	0.092676	0.066408	0.000000	0.000533	0.390768	0.011276	0.052342	0.001882
0.000000	0.133530	0.086014	0.000000	0.001090	0.485514	0.011721	0.044287	0.003559
0.000000	0.086952	0.116440	0.000000	0.000000	0.191823	0.007124	0.074748	0.005612
0.000000	0.166122	0.093035	0.000000	0.000861	0.530700	0.018658	0.037762	0.005528
0.000000	0.081517	0.058845	0.000000	0.000000	0.304758	0.011120	0.029060	0.003655
0.000537	0.097688	0.045396	0.000000	0.000941	0.591805	0.014687	0.035483	0.003377
0.000000	0.093402	0.077117	0.000024	0.000365	0.336166	0.018161	0.021602	0.001992
0.000000	0.091664	0.077348	0.000123	0.001626	0.503285	0.014059	0.043096	0.005347
0.000000	0.098807	0.066470	0.000014	0.000668	0.333558	0.010277	0.038955	0.003237
#NULL!	0.132675	0.050646	#NULL!	0.001018	0.328124	0.015960	0.038764	0.001560
#NULL!	0.210617	0.081473	#NULL!	0.000125	0.348906	0.010699	0.062807	0.005264
#NULL!	0.169547	0.059461	0.000048	0.000222	0.263380	0.012205	0.058806	0.001870
0.000000	0.198448	0.095413	0.000027	0.000047	0.242729	0.010494	0.059333	0.002413
0.000000	0.114282	0.055418	0.000065	0.000780	0.405128	0.015584	0.047674	0.003972
0.000000	0.124129	0.052816	0.000023	0.000843	0.171588	0.010218	0.050848	0.004723
0.000000	0.076271	0.062727	0.000109	0.000672	0.156276	0.008959	0.053849	0.001744

2^-ΔCT	2^-ΔCT	2^-ΔCT	2^-ΔCT
**Ucp2**	**Ucp3**	**Vim**	**Xpa**
0.053484	0.014925	0.104602	0.055091
0.052492	0.018547	0.069543	0.054977
0.052059	0.010504	0.060722	0.037550
0.048218	0.013385	0.071254	0.053047
0.036632	0.011460	0.035899	0.037513
0.056705	0.012695	0.058849	0.051841
0.071155	0.014418	0.052521	0.058555
0.056717	0.014815	0.069110	0.061446
0.065640	0.008990	0.067473	0.059102
0.073986	0.019723	0.066291	0.050778
0.036898	0.012778	0.059893	0.040591
0.033921	0.009263	0.065670	0.047702
0.051474	0.010554	0.077169	0.052500
0.042502	0.008103	0.123781	0.044461
0.035233	0.012677	0.037254	0.036463
0.032239	0.008125	0.081076	0.050224
0.089080	0.015564	0.117183	0.086143
0.034963	0.009407	0.067243	0.051011
1.154825	0.001121	0.190153	0.046117
3.162372	0.000000	0.244429	0.049178
1.015851	0.001215	0.120267	0.061989
4.315630	0.000000	0.291830	0.037628
2.216592	0.000486	0.160095	0.027850
3.244629	0.000441	0.342411	0.030674
2.824344	0.000680	0.371442	0.028692
3.103724	0.000809	0.314449	0.046069
2.358049	0.000000	0.168806	0.051217
1.807126	0.000567	0.209807	0.052129
3.588525	#NULL!	0.252254	0.042072
2.423211	0.000013	0.163537	0.067318
5.822499	0.000050	0.160817	0.038283
1.943864	0.000000	0.241096	0.047898
2.726832	0.000000	0.103009	0.065024
1.820607	0.000024	0.128124	0.050741
