# Supplementary material for: Altered Blood and Brain Expression of Inflammation and Redox Genes in Alzheimer’s Disease, Common to APPV717I × TAUP301L Mice and Patients
Source: Int J Mol Sci. 2022 May 21;23(10):5799. doi: 10.3390/ijms23105799 (PMC9144576; doi:10.3390/ijms23105799)
Supplement: Supplementary file 1 [file ijms-23-05799-s001.zip › Supplementary Table 3.pdf]

Supplementary Table 3. Primer sequences used in this study using qPCR (individual genes).

Gene	Forward primer	Reverse primer
GSR	TGGCACTTGCGTGAATGTTG	TGTTCAGGCGGCTCACATAG
OSGIN1	CGGTGACATCGCCCACTAC	GCTCGGACTTAGCCCACTC
GAPDH	CGACTTCAACAGCAACTCCCACTCTTCC	TGGGTGGTCCAGGGTTTCTTACTCCTT
TBP	TGCACAGGAGCCAAGAGTGAA	CACATCACAGCTCCCCACCA

GSR - Glutathione-Disulfide Reductase;
OSGIN1 - Oxidative Stress Induced Growth Inhibitor 1;
GAPDH - Glyceraldehyde 3-phosphate dehydrogenase;
TBP - TATA-binding protein.
