# Supplementary material for: Altered Blood and Brain Expression of Inflammation and Redox Genes in Alzheimer’s Disease, Common to APPV717I × TAUP301L Mice and Patients
Source: Int J Mol Sci. 2022 May 21;23(10):5799. doi: 10.3390/ijms23105799 (PMC9144576; doi:10.3390/ijms23105799)
Supplement: Supplementary file 1 [file ijms-23-05799-s001.zip › Supplementary Table 4.pdf]

Supplementary Table 4. Comparison of genes dysregulated in the hippocampus of AT mice (FC>|2|, p<0.05) and in post-mortem samples of AD brain from different microarray data sets considering a FR>|1.3| and nominal p-value < 0.05.

	**Gene**	**AT *vs* WT (FC>|2|, HP)**	**GEODATASET Human Brain**	**Significance**	**Ref**
**Inflammatory genes**	**IL-19**	↑ FR=3.66; p=0.034	↑ 17 AD vs 19 CTRL (TB)	FR=1.40; p=0.0004*	[GSE138260]
			↑ 7 AD vs 4 CTRL (FC)	**FR=1.80**; p=0.03	[GSE185909]
	**F2R**	↑ FR=2.84; p=0.004	↑ 7 incipient AD vs 9 CTRL (HP)	**FR=2; p=0.004**	[GSE1297]
			↑ 7 severe AD vs 9 CTRL (HP)	**FR=2.03; p=0.002**	[GSE1297]
			↑ 36 AD vs 16 CTRL (EC)	FR=1.38; p=0.0012*	[GSE118553]
	**TLR6**	↑ FR=2.75; p=0.001	↑ 7 AD vs 4 CTRL (FC)	**FR=1.62; p=0.024**	[GSE185909]
	**IL-1B**	↑ FR=2.90; p=0.006	↓ 7 severe AD vs 8 CTRL (GM)	FR=-10.86; p=0.00000698	[GSE28146]
	**LTA**	↑ FR=2.17; p=0.034	↑ 17 AD vs 19 CTRL (TB)	FR=1.49; p=0.001*	[GSE138260]
			↑ 7 AD vs 4 CTRL (FC)	**FR=1.61; p=0.024**	[GSE185909]
			↑7 incipient AD vs 8 CTRL (GM)	**FR=3.51; p=0.002**	[GSE28146]
			↑ 8 AD vs 8 CTRL (NCX)	**FR=1.57; p=0.05**	[GSE37264]
	**CSF2**	↓ FR=-2.20; p=0.021	↑ 7 incipient AD vs 9 CTRL (HP)	FR=1.98; p=0.02	[GSE1297]
			↑ 7 severe AD vs 9 CTRL (HP)	FR=2.41; p=0.00009546	[GSE1297]
**Redox genes**	**RAG2**	↑ FR=3.80; p=0.034	↑ 19 AD vs 26 CTRL (HP)	FR=1.33; p=0.00453	[GSE48350]
	**NOXA1**	↑ FR=3.39; p=0.009	↓ 17 AD vs 19 CTRL (TB)	FR=-1.34; p=0.001*	[GSE138260]
	**MPO**	↑ FR=2.64; p=0.034	↑ 7 AD vs 4 CTRL (FC)	FR=1.336; p=0.029	[GSE185909]

	**GPX3**	↑ FR=2.36; p=0.016	↑ 36 AD vs 16 CTRL (EC)	**FR=1.66; p=0.000025***	[GSE118553]
	**CYBA**	↑ FR=2.21; p=0.012	↑ 36 AD vs 18 CTRL (CRB)	FR=1.41; p=0.0175*	[GSE118553]
			↑ 36 AD vs 16 CTRL (EC)	FR=1.31; p=0.018*	[GSE118553]
	**EPX**	↑ FR=2.15; p=0.009	↑ 7 AD vs 4 CTRL (FC)	FR=1.42; p=0.022	[GSE185909]
			↓ 36 AD vs 16 CTRL (EC)	FR=-1.37; p=0.01*	[GSE118553]
	**NOX1**	↑ FR=2.14; p=0.004	↑ 7 incipient AD vs 9 CTRL (HP)	**FR=1.66; p=0.04**	[GSE1297]

Legend: HP= hippocampus; FCX= frontal cortex; GM= grey matter; NCX=neocortex; EC= entorhinal cortex; CRB=cerebellum; TB=total brain; *adjusted p-value<0.05
